# Patients‐Derived Organoids Sequencing‐based FOXP4 Facilitates Radioresistance by Transcriptionally Modifying GPX4 to Regulate ferroptosis in Colorectal Cancer

**DOI:** 10.1002/advs.202507080

**Published:** 2025-08-11

**Authors:** Qianping Chen, Yuzhao Jin, Luyu Liao, Rong Shen, Xingnan Ge, Qingyu Jiang, Ying Huang, Quanquan Sun, Dong Liu, Luying Liu, Tongxin Liu, Qinghui Dai, Xiyuan Tang, Zhe Han, Xin Gao, Xinhua Lin, Wei Mao, Ji Zhu

**Affiliations:** ^1^ Department of Abdominal Radiotherapy Zhejiang Cancer Hospital Hangzhou 310022 P. R. China; ^2^ Hangzhou Institute of Medicine (HIM) Chinese Academy of Sciences Hangzhou 310022 P. R. China; ^3^ Wenzhou Medical University Wenzhou 325000 P. R. China; ^4^ Zhejiang Chinese Medicine University Hangzhou 310053 P. R. China; ^5^ Orthopaedic Oncology Center Department of Orthopedics Changzheng Hospital Naval Medical University Shanghai 200003 P. R. China; ^6^ School of Basic Medical Sciences Lanzhou University Gansu 730000 P. R. China; ^7^ Key Laboratory of Virology and Biosafety Wuhan Institute of Virology Chinese Academy of Sciences Wuhan 430200 P. R. China; ^8^ School of Life Sciences Fudan University Shanghai 200438 P. R. China

**Keywords:** Ferroptosis, FOXP4, GPX4, PDOs, Radioresistance

## Abstract

More than 50% of patients with colorectal cancer (CRC) exhibit radioresistance, indicating the need for further research on the disease. Therefore, the aim of this study is to identify radioresistance genes and elucidate the underlying molecular mechanisms using patient‐derived organoids (PDOs). Transcriptome analyses are performed on radio‐resistant and ‐sensitive PDOs, CRC cells, and xenograft tissues to screen for radioresistant genes. Additionally, the genetic homology between PDOs and clinical tissues is verified using whole‐exome sequencing. Functional experiments are performed to validate the roles of the candidate genes using cellular, organoid, and animal models. Forkhead box P4 (FOXP4) is identified as a differentially expressed genes between the radio‐sensitive and ‐resistant groups that is linked to radioresistance. Further experiments show that FOXP4 promoted radioresistance by suppressing ferroptosis. Mechanistically, FOXP4 regulated GPX4 transcription by binding to the promoter region of GPX4 via the forkhead domain to inhibit the onset of ferroptosis. Doxorubicin (DOX) inhibited FOXP4 expression by promoting its ubiquitination and degradation, eventually increasing radiosensitivity. Notably, DOX combined with irradiation attenuated the compensatory increase in FOXP4 expression and increased radiotherapy efficacy. Conclusively, the combination therapy provides a new strategy for enhancing therapeutic efficacy in CRC.

## Graphical abstract

FOXP4 regulates GPX4 transcription to suppress ferroptosis. In CRC patients, initial biopsies established PDO cultures alongside concurrent chemoradiotherapy. Post‐treatment MRI with mrTRG scoring stratified radioresistant/sensitive cohorts. Parallelly, 8Gy‐irradiated PDOs underwent viability‐based stratification. Integrating clinical outcomes and PDO results, multi‐omics sequencing identified FOXP4. Mechanistically, doxorubicin (DOX) promoted FOXP4 ubiquitinated degradation, inhibiting GPX4 transcription to induce ferroptosis and enhance CRC radiosensitivity.

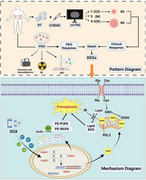

## 1 Introduction

Colorectal cancer (CRC) is the third most prevalent malignancy and second leading cause of cancer‐related mortality worldwide.^[^ ^1^ ^]^ Currently, the standard treatment involves preoperative neoadjuvant chemoradiotherapy followed by total mesorectal excision and postoperative adjuvant chemotherapy, particularly for patients with locally advanced rectal cancer (LARC).^[^ ^2^ ^]^ Although this approach has improved local control and survival rates, the complete remission (CR) rate is only 15%.^[^ ^3^ ^]^ In our previous multicenter phase III clinical study (ClinClare), we successfully increased the CR rate in rectal cancer to 30% by combining irinotecan and capecitabine (CapIriRT regimen) during synchronous radiotherapy.^[^ ^4^ ^]^ However, many patients are not susceptible to CapIriRT regimens, highlighting the need to elucidate the mechanisms underlying radioresistance.

A previous study showed that insufficient ferroptosis activation was one of the main mechanisms underlying radioresistance in hepatocellular carcinoma cells.^[^ ^5^ ^]^ As major types of gastrointestinal tumors, CRC and hepatocellular carcinoma are correlated in terms of pathogenesis and treatment prognosis. Since Fe^2+^ is predominantly reabsorbed in the intestine, investigating ferroptosis in CRC radioresistance holds significant value. Ferroptosis is an iron‐dependent and lipid peroxidation‐driven form of programmed cell death.^[^ ^6^ ^]^ High Fe^2+^ and reactive oxygen species (ROS) levels induce the peroxidation of phospholipids containing polyunsaturated fatty acids, resulting in the accumulation of lipid peroxides, eventual rupture of the cell membrane, and cell death.^[^ ^7^ ^]^ Irradiation promotes ferroptosis by generating large amounts of ROS, leading to lipid peroxide accumulation.^[^ ^8^ ^]^ Ferroptosis inducers, such as erastin and RSL3, have been identified as potential therapeutic agents for cancer. Notably, these compounds function by inhibiting the systems that protect cells from oxidative damage, thereby promoting ferroptosis in cancer cells.^[^ ^9^ ^]^ Ferroptosis agonists can inhibit tumor progression and overcome radiotherapy resistance; therefore, inducing ferroptosis could be a potential way to optimize neoadjuvant therapy.^[^ ^10^ ^]^

The Forkhead box P (FOXP) family of transcription factors, including FOXP1, FOXP2, FOXP3, and FOXP4, plays significant roles in various biological processes, including development and immune regulation.^[^ ^11^ ^]^ FOXP1 is implicated in the progression of several cancers, including CRC. FOXP1 overexpression promotes malignant progression and facilitates immunosuppression by reprogramming the glycolytic metabolism in cervical cancer.^[^ ^12^ ^]^ Research has shown that high FOXP2 expression predicts poor survival and facilitates the proliferation and differentiation of prostate cancer cells by mobilizing mesenchymal to epithelial transition factor signaling.^[^ ^13^ ^]^ FOXP3 is best known for promoting immune escape and suppressing antitumor immunity as a Treg cell marker and is strongly associated with poor prognosis in breast and colorectal cancer.^[^ ^14^ ^]^ Similar to its family members, FOXP4 consists of an N‐terminal domain, a zinc‐finger domain, and a highly conserved forkhead (FH) domain at the C‐terminal end.^[^ ^15^ ^]^ FOXP4 may function as an oncogene in various cancer types, and has been identified as a direct target of the YAP1 signaling pathway to maintain cancer stemness and promote metastasis in gastric cancer.^[^ ^16^ ^]^ Recent research have revealed that FOXP4 modulates tumor growth in ovarian cancer and is independently associated with patient prognosis.^[^ ^17^ ^]^ However, the role of FOXP4 in CRC remains unclear.

Based on the research gap, the aim of this study was to identify radioresistance genes and elucidate the underlying molecular mechanisms using patient‐derived organoids (PDOs), CRC cell lines, and xenograft models. Transcriptome analysis was performed to identify radioresistance genes in CRC, followed by functional experiments to validate the roles of the identified genes in radioresistance in CRC. Overall, it is anticipated that this study will provide new insights into the molecular mechanisms underlying CRC radioresistance and potential therapeutic targets to improve treatment outcomes.

## 2 Result

### 2.1 FOXP4 Shows Significant Correlation with Radioresistance in CRC

**Figure 1:**
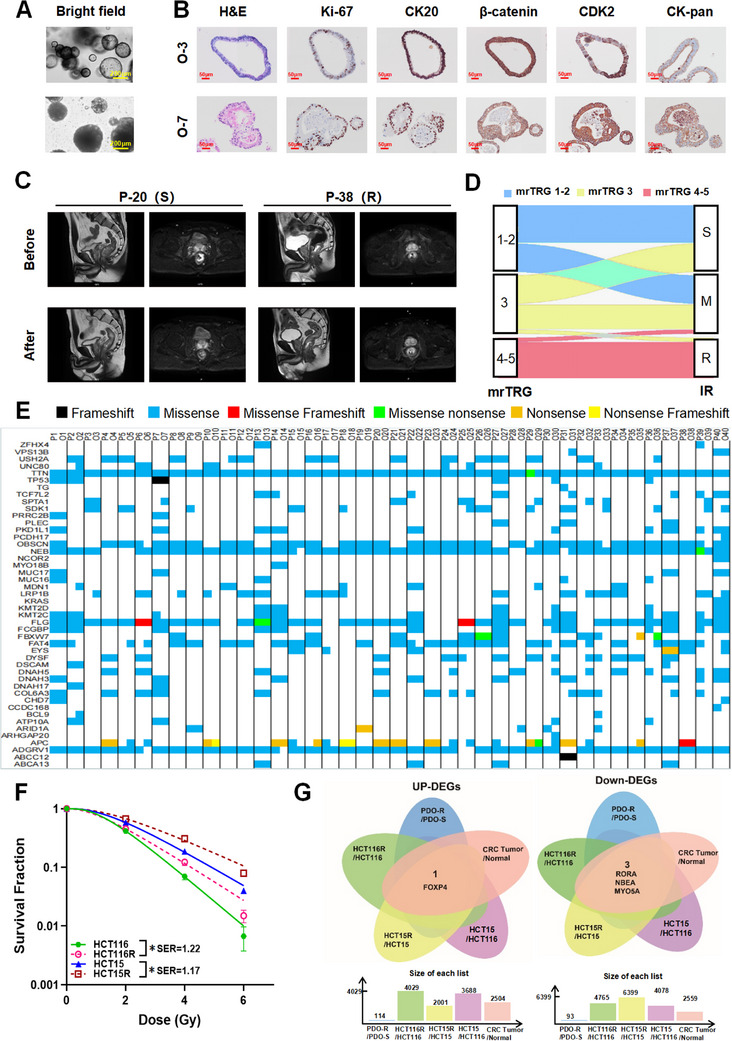
FOXP4 was significantly associated with radioresistance in colorectal cancer. A) The morphology of PDOs with two typical characteristics in bright field (walled cystic structure and solid spherical structure). Scale bar, 200 µm. B) H&E staining and IHC staining of Ki‐67, CK20, β‐catenin, CDX2 and CK‐pan on typical structure CRC PDO (O3: walled cystic structure; O7: solid spherical structure, O refers to PDO). Scale bar, 50 µm. C) Typical MRI images of patients with significant tumor regression (mrTRG 1‐2, P‐20) and patients with insignificant tumor regression (mrTRG 4‐5, P‐38) after radiotherapy, P refers to Patient. D) The mrTRG score of patients after radiotherapy matched organoids survival levels after IR, S refers to radiosensitive PDO, R refers to radioresisitant PDO, M refers to medium response to irradiation. E) Heatmap of gene mutation variations (single‐nucleotide variants, SNVs) in the most frequently mutated genes of rectal cancer. F) Dose responses of survival fractions of HCT116, HCT116R, HCT15 and HCT15R after IR. G) Venn diagram of the co‐expressed DEGs among five groups (Green: HCT116R/HCT116; Yellow: HCT15R/HCT15; Purple: HCT15/HCT116; Blue: PDO‐R/PDO‐S; Pink: Tumor/Normal tissues). ^*^ *P* ＜ 0.05, ^**^ *P* ＜ 0.01, ^***^ *P* ＜ 0.001.

In this study, a magnetic resonance (MR) examination was performed to clarify the baseline tumor stage in patients with rectal cancer, followed by anoscopy to collect rectal cancer tissues that were initially diagnosed without any treatment. Additionally, we generated 96 CRC PDOs and performed light microscopy, hematoxylin and eosin (H&E) staining, and immunohistochemical (IHC) assay to clarify the biological and pathological characteristics of the organoids (**Figure** **1**A,B).

In total, 96 patients were treated with neoadjuvant radiotherapy first (45‐50.4 Gy/25‐28F radiotherapy concurrently with receiving a synchronous chemotherapy regimen of irinotecan and capecitabine) and subjected to MR after the completion of the synchronous radiotherapy to assess tumor regression. Additionally, the MR results after simultaneous radiotherapy were compared with the baseline MR data to obtain the mrTRG score. Patients with mrTRG scores of 1–2 were classified as radiosensitive, whereas those with scores of 4–5 were defined as radioresistant (Figure 1C). Among the 96 patients, 39.6%, and 22.9% were classified as radio‐sensitive and ‐resistant, respectively, as of August 2024 (Tables S1 and S2, Supporting Information). Additionally, we performed irradiation experiments on all 96 organoids and ranked them into three categories based on survival. Notably, the first category with 39.6% survival rate was the radiosensitive group, whereas the last category with 22.9% survival rate was the radioresistant group (Tables S1 and S2, Supporting Information). Thereafter, we matched the patients' clinical radiosensitivity with organoid radiosensitivity and identified 20 radiosensitive and 20 radioresistant groups (Figure 1D). Moreover, whole‐exosome sequencing was performed to ensure genetic consistency between the PDOs and clinical samples (Figure 1E; Figure S1A and Table S3, Supporting Information).

Furthermore, we generated the acquired radioresistant CRC cell lines HCT116R and HCT15R via consistent irradiation of HCT116 and HCT15 cells at 50 Gy (2 × 25 Gy). Dose survival curves after irradiation at 0, 2, 4, and 6 Gy demonstrated a sequential increase in radioresistance in HCT116, HCT116R, HCT15, and HCT15R cells. Among the cell lines, the sensitivity enhancement ratios (SER) of HCT116R/HCT116 and HCT15R/HCT15 were 1.22 and 1.17, respectively (Figure 1F). Importantly, differences in radioresistance among the cell lines were also confirmed using cell viability assay (Figure S1B, Supporting Information).

RNA sequencing was performed on organoids and CRC cells to identify key differentially expressed genes (DEGs) involved in radioresistance. Notably, DEGs between the radio‐resistant and ‐sensitive PDOs, HCT116R and HCT116 cells, HCT15R and HCT15 cells, and HCT15 and HCT116 cells are shown in Figure S1C–F (Supporting Information). Additionally, we screened DEGs in CRC tissues and paracancerous intestinal epithelial tissues from the TCGA database (Figure S1G, Supporting Information). Based on the intersection of the DEGs, the upregulated gene FOXP4 and three downregulated genes (RORA, NBEA, and MYO5A) were identified as potential regulatory genes for CRC radiotherapy (Figure 1G).

Considering that highly expressed genes are more convenient for designing corresponding antibodies or inhibitors for clinical translation, we verified the regulatory role of FOXP4 in CRC radiosensitivity. FOXP4 was highly expressed in CRC tissues compared with that in adjacent normal tissues in the GEPIA database and was associated with poor prognosis across multiple databases, suggesting its potential as a target for screening antitumor therapeutic sensitizing drugs (Figure S1H–J, Supporting Information).

### 2.2 FOXP4 Modulates Radiosensitivity in CRC both In Vivo and In Vitro

**Figure 2:**
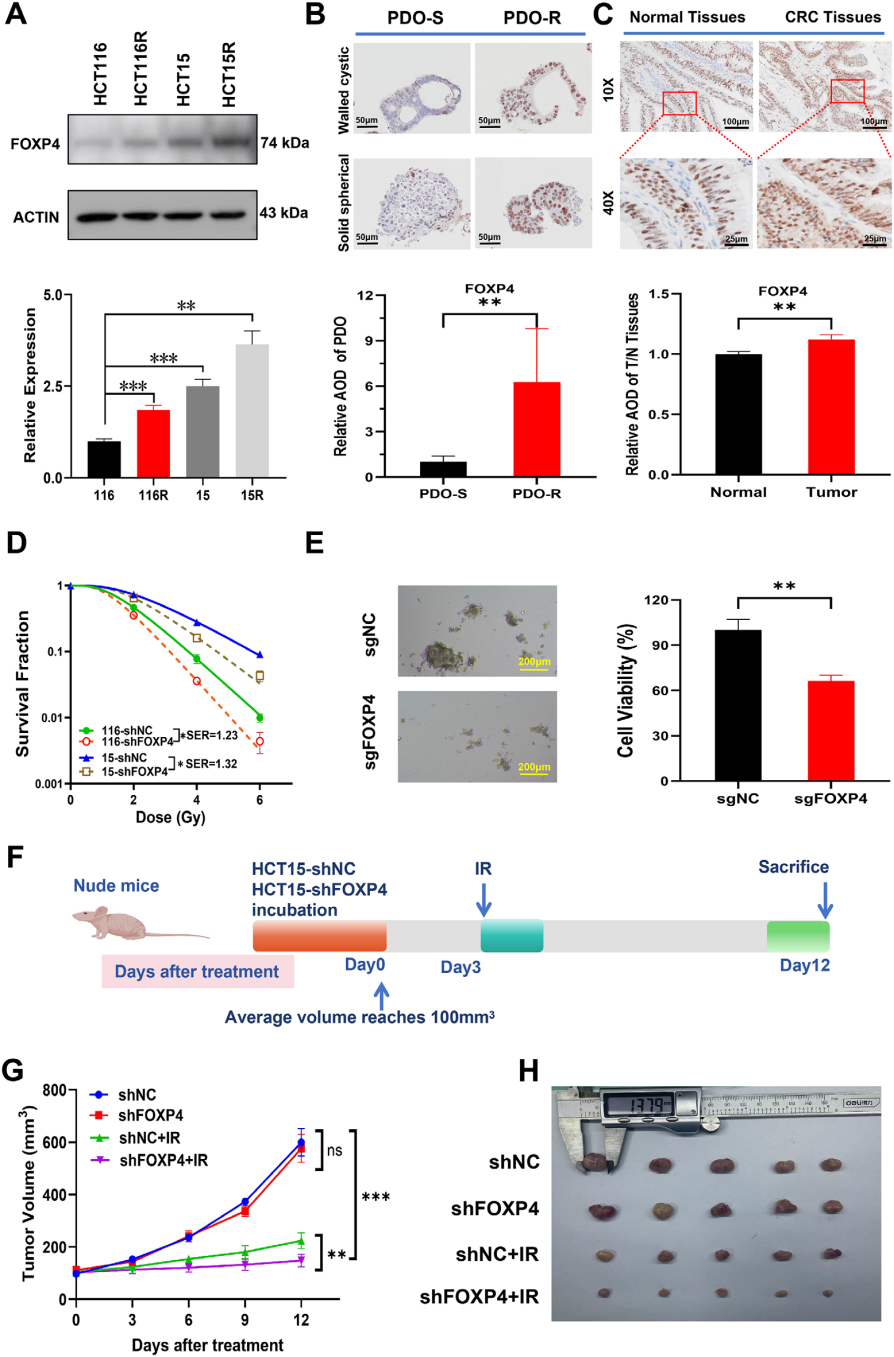
Inhibition of FOXP4 significantly enhanced the radiosensitivity of colorectal cancer. A) Representative images and quantification of western blot assay of FOXP4 and β‐actin proteins in HCT116, HCT116R, HCT15 and HCT15R cells. B) Representative images and quantification of IHC staining of FOXP4 on radiosensitive and radioresistant PDOs. *n*=5. Scale bar, 50 µm. C) Representative images and quantification of IHC staining of FOXP4 on CRC tumor tissues and adjacent normal tissues. *n*=3. 10X Scale bar, 100 µm; 40X Scale bar, 25 µm. D) Dose responses of survival factions of HCT116 and HCT15 cells with or without shFOXP4 transfection. E) Bright field image and quantitative results of PDO transfected with sgFOXP4 or sgNC after 8Gy irradiation. *n*=5. Scale bar, 200 µm. F) Pattern plots of nude mice inoculated with shNC or shFOXP4 HCT15 cells that were irradiated at 8Gy*3d and then executed at the appropriate time. G) Tumor volume of shNC, shFOXP4, shNC+IR and shFOXP4+IR groups was examined every 3 days until 9 days after IR. H) General view of tumor mass of each indicated group at 9 days after IR.^*^ *P* ＜ 0.05,^**^ *P* ＜ 0.01,^***^ *P* ＜ 0.001.

To verify the reliability of the bioinformatic results, we examined FOXP4 expression at the cellular, organoid, and clinical tissue levels. FOXP4 expression increased gradually in HCT116, HCT116R, HCT15, and HCT15R (**Figure** **2**A). Additionally, IHC assay showed that FOXP4 expression was higher in radioresistant PDOs and CRC tissues than in radiosensitive PDOs and paracancerous tissues, respectively (Figure 2B,C). shRNA‐mediated FOXP4 knockdown in CRC cells significantly enhanced radiosensitivity, with SER values of 1.23 and 1.32, respectively (Figure 2D). Similarly, sgRNA‐mediated FOXP4 inhibition in PDOs resulted in a higher mortality rate following irradiation at 8 Gy (Figure 2E). To investigate the role of FOXP4 in vivo, HCT15 cells transfected with shFOXP4 were subcutaneously injected into nude mice. At a size of 100 mm^3^, the transplanted tumors were irradiated for 3 days at 8 Gy (Figure 2F). Notably, both the growth curves and tumor images indicated that FOXP4 inhibition increased the radiosensitivity of CRC cells in vivo (Figure 2G,H). Collectively, these results indicate that FOXP4 inhibition/knockdown increases CRC radiosensitivity at the cellular, organoid, and animal levels.

### 2.3 Ferroptosis Activation Induces Radiosensitization in CRC

**Figure 3:**
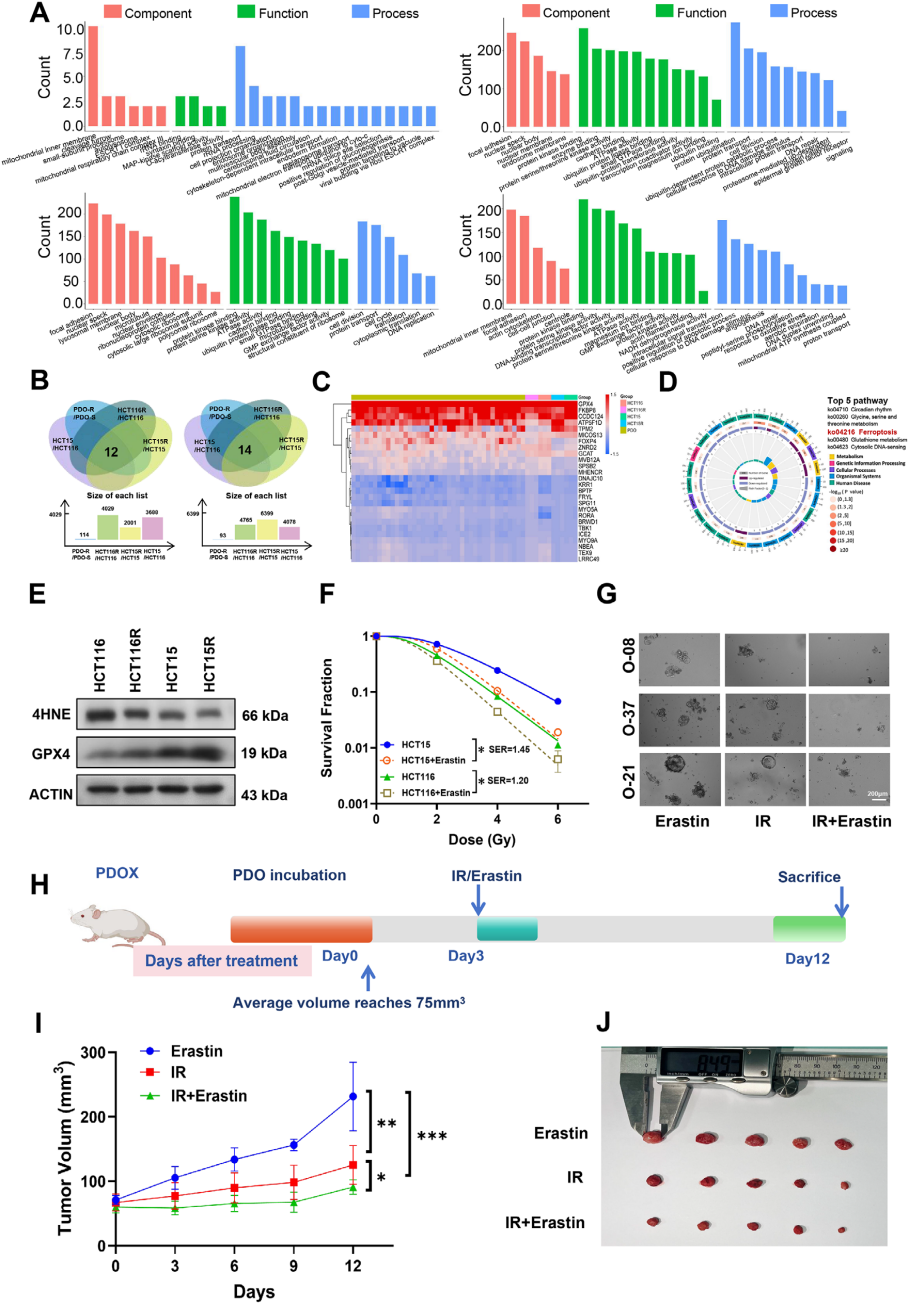
Enhanced ferroptosis resulted in radiosensitization of colorectal cancer. A) GO analysis of DEGs from PDO‐R vs PDO‐S, HCT116R vs HCT116, HCT15R vs HCT15, and HCT15 vs HCT116. Fold change ≥ 1.5 or ≤ 0.67, *P*‐value < 0.01; B) Venn diagram of the co‐expressed DEGs among above four groups (Blue: PDO‐R/PDO‐S, Green: HCT116R/HCT116, Yellow: HCT15R/HCT15, Purple: HCT15/HCT116). C) Heatmap of co‐expression of DEGs in the above four groups (PDO‐R/PDO‐S, HCT116R/HCT116, HCT15R/HCT15, HCT15/HCT116). D) Pathway enrichment analysis of co‐expressed DEGs in the above four groups (PDO‐R/PDO‐S, HCT116R/HCT116, HCT15R/HCT15, HCT15/HCT116). E) Western blot assay of 4HNE, GPX4, and β‐actin proteins in HCT116, HCT116R, HCT15, and HCT15R cells. F) Dose responses of survival fractions of HCT116 and HCT15 cells with or without Erastin treatment after IR. G) Bright field images of PDOs treated with Erastin or 8Gy IR. Scale bar, 200 µm. H. Pattern plots of PDOX treated with Erastin or not that were irradiated at 8Gy*3d and then executed at the appropriate time. I) Tumor volume of Erastin, IR, and Erastin+IR groups was examined every 3 days until 9 days after IR. J) General view of tumor mass of each indicated group at 9 days after IR. ^*^ *P* ＜ 0.05, ^**^ *P* ＜ 0.01,^***^ *P* ＜ 0.001.

To investigate the mechanism regulating radiosensitivity in CRC, we performed bioinformatics analysis of the sequencing datasets, PDO‐R/PDO‐S, HCT116R/HCT116, HCT15R/HCT15, and HCT15/HCT116. Additionally, the gene ontology (GO) analysis plots for the four sets of sequencing data are shown in **Figures** **3**A and S2 (Supporting Information). Moreover, Kyoto Encyclopedia of Genes and Genomes (KEGG) analysis indicated that 26 DEGs involved in radiosensitivity regulation were enriched in pathways such as ferroptosis, circadian rhythm and glycine, serine and threonine metabolism (Figure 3B–D).

Considering that ferroptosis has been shown to play an important role in CRC occurrence and development, we investigated whether ferroptosis participates in mediating radiosensitization in CRC. Therefore, we examined the expression of 4‐hydroxynonenal (4HNE), a key biomarker of ferroptosis, and GPX4, a negatively regulated protein, in the cell lines after irradiation at 4 Gy. Notably, ferroptosis activation decreased with increasing radioresistance (Figure 3E; Figure S3A, Supporting Information). Therefore, we hypothesized that insufficient ferroptosis activation is a key factor contributing to radioresistance in CRC.

To verify the hypothesis that ferroptosis activation can enhance CRC radiosensitivity, CRC cells were treated with the ferroptosis agonist erastin. Importantly, the dose survival curves showed that erastin significantly improved the radiosensitivity of CRC cells by activating ferroptosis (Figure 3F). Additionally, cell viability assay using the Cell Counting Kit‐8 (CCK‐8) confirmed that irradiation (4 Gy) combined with erastin had a higher inhibitory effect on the growth of CRC cells than irradiation or erastin alone (Figure S3B, Supporting Information). At the organoid level, irradiation combined with erastin significantly reversed radioresistance in CRC organoids (Figure 3G; Figure S3C, Supporting Information).

Furthermore, in vivo experiments were performed to determine whether ferroptosis activation can improve radiosensitivity in mice with CRC. Specifically, a patient‐derived organoid‐based xenograft (PDOX) model was constructed by subcutaneously inoculating fully immunodeficient mice with PDOs. Drug treatment and irradiation were administered following tumor growth to an appropriate size (Figure 3H). Both growth curves and tumor mass images demonstrated that erastin improved the efficacy of radiotherapy in vivo (Figure 3I,J).

Compared with that in the erastin group, 4HNE expression was further upregulated in the IR group (Figure S3D, Supporting Information). Irradiation combined with erastic treatment significantly activated ferroptosis pathways, indicating that erastin had a significant radiosensitization effect. However, in addition to activating ferroptosis, FOXP4 expression was upregulated in the IR group. In contrast, irradiation combined with erastin treatment inhibited FOXP4 expression and further induced ferroptosis (Figure S3D, Supporting Information). Nude mice were subcutaneously inoculated with HCT15 cells to construct a transplanted tumor system. After growing to a suitable size, the transplanted tumors in the nude mice were subcutaneous irradiation alone, treated with erastin alone, or irradiated and treated with erastin (Figure S3F, Supporting Information). Consistent with our findings in the organoids model, the combination of irradiation and erastin significantly inhibited the growth rate and volume of the transplanted tumors compared with irradiation or erastin treatment alone (Figure S3G,H, Supporting Information). Compared with those in the IR group, IHC assay confirmed that the ferroptosis marker protein 4HNE was significantly upregulated and FOXP4 expression was significantly downregulated in the irradiation and erastin co‐treatment group (Figure S3E, Supporting Information). Based on these results, we speculate that irradiation‐induced cell death partially depends on the activation of ferroptosis. Irradiation not only activates ferroptosis but also upregulates FOXP4, limiting antitumor efficacy. However, irradiation combined with erastin inhibits the expression of FOXP4, thereby amplifying the occurrence of ferroptosis and increasing irradiation‐induced cell death.

### 2.4 FOXP4 Induces Ferroptosis both In Vivo and In Vitro

**Figure 4:**
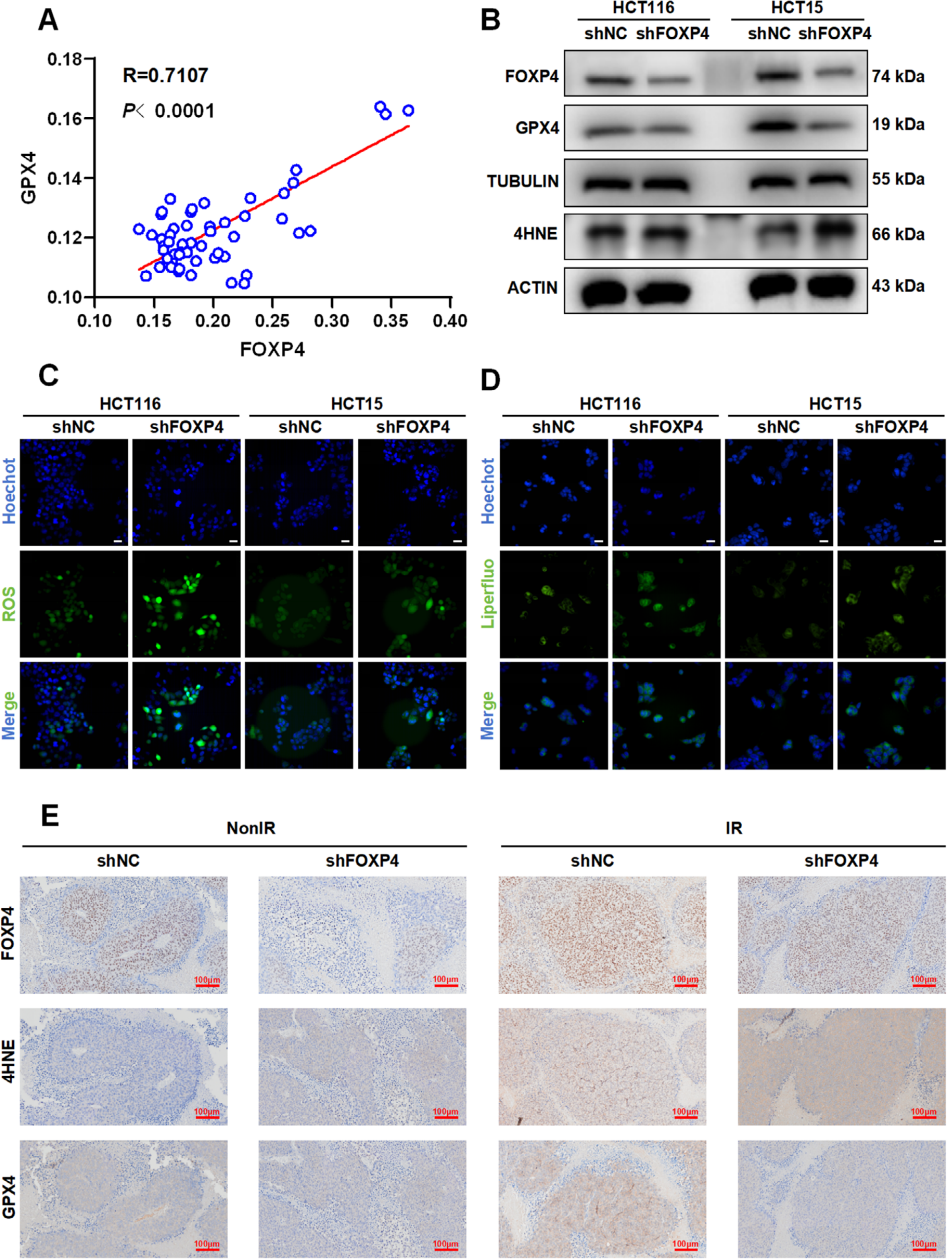
FOXP4 regulated ferroptosis in CRC. A) Scatter diagrams showed the correlation between FOXP4 and GPX4 in organoids and cell lines. B) Western blot assay of FOXP4, 4HNE, GPX4, β‐actin and α‐tubulin proteins in HCT116 and HCT15 cells transfected with shNC or shFOXP4. C) Representative images of ROS in HCT116 and HCT15 cells transfected with shNC or shFOXP4 at 4h after 4Gy IR. Nuclei were stained with Hoechst (×40). Scale bars, 10 µm. D) Representative images of liperfluo (a lipid peroxide fluorescent probe) in HCT116 and HCT15 cells transfected with shNC or shFOXP4 at 4h after 4Gy IR. Nuclei were stained with Hoechst (×40). Scale bars, 10 µm. E) Representative IHC images (x20) of FOXP4, 4HNE and GPX4 protein expression of nude mice xenograft tumors. Scale bar, 100 µm. ^*^ *P* < 0.05, ^**^ *P* < 0.01 and ^***^ *P* < 0.001.

Considering that FOXP4 inhibition increases radiosensitivity in CRC and ferroptosis activation reverses radioresistance in CRC, we hypothesized that there is a reciprocal regulation between ferroptosis and FOXP4 protein. As shown in Figure 3C, GPX4, a key protein of ferroptosis, was involved in regulating sensitivity to radiotherapy in CRC and displayed the highest level of differential expression in the four sequencing datasets. Additionally, correlation analysis indicated that FOXP4 was strongly correlated with GPX4 in CRC cells and organoids, with a correlation coefficient of 0.8109 (**Figure** **4**A). Therefore, we examined the effect of FOXP4 expression on GPX4‐regulated ferroptosis. FOXP4 inhibition significantly downregulated GPX4 expression and significantly upregulated 4HNE expression compared with those in the control group (Figure 4B, Figure S4A, Supporting Information). Overall, these results indicate that FOXP4 inhibition may promote ferroptosis activation at the protein level. Research findings indicate that ferroptosis occurs through increased lipid peroxidation and elevated ROS levels. Therefore, we examined changes in ROS and lipid peroxide levels in CRC cells following FOXP4 inhibition. Intracellular reduction of FOXP4 expression effectively upregulated ROS levels in the cells (Figure 4C; Figure S4B, Supporting Information). Additionally, analysis of intracellular lipid peroxide levels using Liperfluo and malondialdehyde (MDA) assays showed that FOXP4 inhibition significantly enhanced Liperfluo accumulation in cells (Figure 4D; Figure S4C, Supporting Information) and intracellular MDA content (Figure S4D, Supporting Information).

To confirm the role of FOXP4 on ferroptosis in vivo, nude mice were subcutaneously transplanted with FOXP4 knockdown and non‐knockdown tumors (Figure 2F‐H), followed by the assessment of FOXP4, GPX4, and 4HNE expression (Figure 4E; Figure S4E, Supporting Information). FOXP4 inhibition markedly suppressed GPX4 expression and enhanced 4HNE accumulation, thereby triggering ferroptosis in vivo to induce cell death. Notably, this effect was more pronounced after irradiation.

### 2.5 FOXP4 Knockdown Sensitizes CRC Cells to Radiotherapy by Promoting Ferroptosis

**Figure 5:**
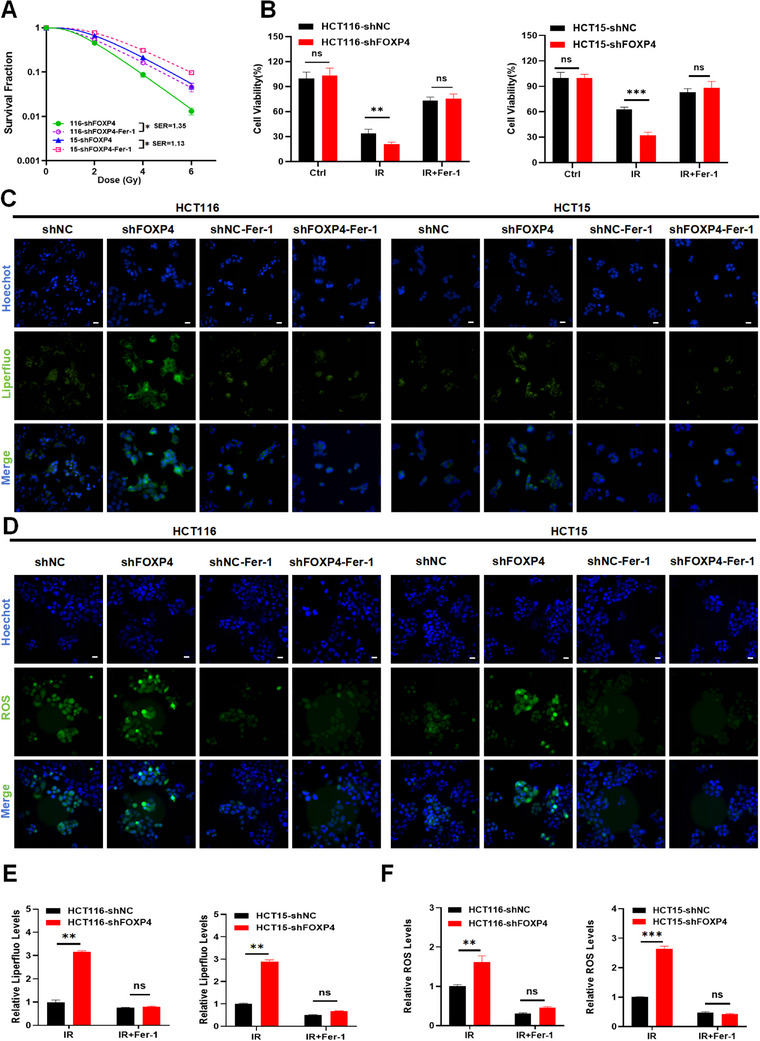
FOXP4 induced radioresistance by blocking ferroptosis in CRC cells. A) Dose responses of survival factions of HCT116‐shFOXP4 and HCT15‐shFOXP4 cells treated with Fer‐1. B. CCK‐8 assay measured cell viability of HCT116 and HCT15 cells at 48h after 4Gy IR or Fer‐1 treatment. C,E) Representative images (×40, C) and quantification (E) of liperfluo in HCT116 and HCT15 cells transfected with shNC or shFOXP4 and treated with Fer‐1 simultaneously at 4h after 4Gy IR. Nuclei were stained with Hoechst (×40). Scale bars, 10 µm. D–F) Representative images (×40, D) and quantification (F) of the relative fluorescence intensity of ROS in HCT116 and HCT15 cells transfected with shNC or shFOXP4 and treated with Fer‐1 simultaneously at 4h after 4Gy IR. Nuclei were stained with Hoechst (×40). Scale bars, 10 µm. ^*^ *P* ＜ 0.05, ^**^ *P* ＜ 0.01,^***^ *P* < 0.001.

To determine whether the radio‐sensitizing ability of FOXP4 was caused by ferroptosis, we designed a series of rescue experiments. Specifically, we examined the effect of FOXP4 inhibition combined with Ferrostatin‐1 (Fer‐1) treatment (ferroptosis inhibitor) on the survival of CRC cells under irradiation through clone formation experiments. Previous dose‐survival curves revealed that FOXP4 inhibition decreased cell survival after radiation. In contrast, Fer‐1 treatment exerted a radioprotective effect and markedly improved the survival of sh‐FOXP4 CRC cells after radiation (**Figure** **5**A). Similarly, CCK‐8 assay indicated that Fer‐1 treatment enhanced the viability of CRC cells after irradiation at 4 Gy (Figure 5B). Additionally, we examined the modulatory effect of Fer‐1 treatment on other indicators of ferroptosis and found that cells with low FOXP4 expression (HCT116‐shFOXP4, HCT15‐ shFOXP4) had higher intracellular ROS and lipid peroxide levels than HCT116 and HCT15 cells S4F,G. FOXP4 inhibition significantly activated ferroptosis in CRC, whereas Fer‐1‐mediated ferroptosis inhibition significantly decreased intracellular ROS, Liperfluo, and MDA levels (Figure 5C–F; Figure S4F,G, Supporting information). Overall, these results suggest that FOXP4 knockdown increases ferroptosis, whereas Fer‐1 treatment inhibits ferroptosis and restores the radioresistance in CRC cells with low FOXP4 expression.

### 2.6 FOXP4 Acts as a Transcription Factor for GPX4

**Figure 6:**
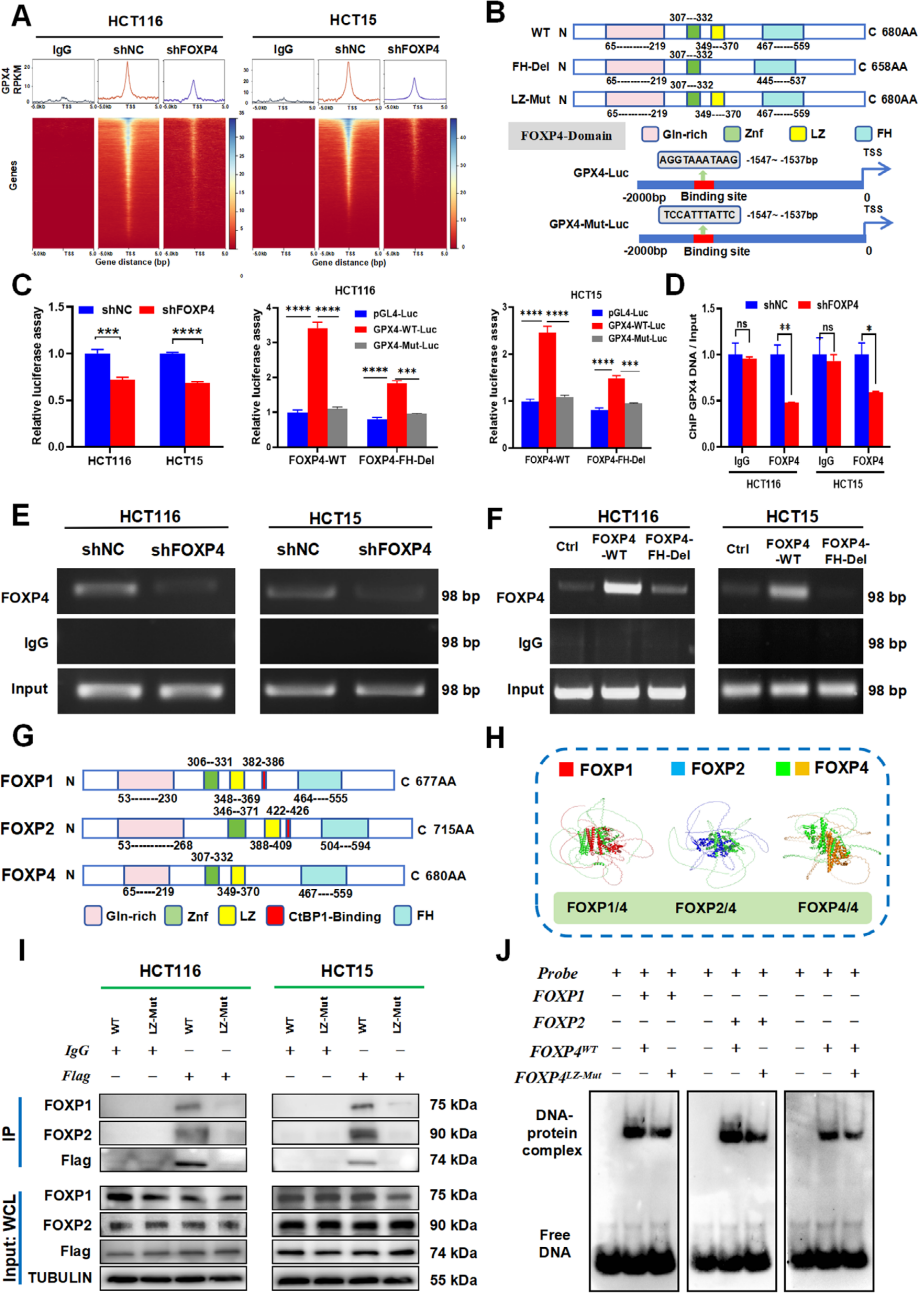
FOXP4 promoted GPX4 transcription in a dimerized form. A) Levels of GPX4 bound at the TSS of peaks in HCT116 and HCT15 cells in CUT&Tag‐seq analysis. Transcription start site, TSS. B. Cartoon diagram showed structures of FOXP4 (WT, FH‐Del and LZ‐Mut), GPX4‐Luc and GPX4‐MUT‐Luc. C) Dual‐luciferase reporter assay showed relative luciferase expression after knockdown of FOXP4 in HCT15 and HCT116 cells. *n* = 3. D–E) Quantification data (D) and agarose gel images (E) of ChIP PCR in HCT116 and HCT15 cells with FOXP4 knockdown. *n* = 3. F) Agarose gel images of ChIP PCR in HCT116 and HCT15 cells with overexpression of FOXP4‐WT or FOXP4‐FD‐Del. G) Cartoon diagram showed structures of FOXP1, FOXP2, and FOXP4. H) Cartoon diagram of FOXP4 dimerization with FOXP1, FOXP2 and FOXP4 by AlphaFold 2. I) Co‐IP assay of FOXP1 or FOXP2 and FOXP4‐WT or FOXP4‐LZ‐Mut in the whole cell lysates of HCT116 and HCT15 cells. J) EMSA measured the interaction between probes compassing the GPX4 motif and different protein (FOXP4‐WT with FOXP1, FOXP2 or FOXP4‐LZ‐Mut).^*^ *P* ＜ 0.05, ^**^ *P* ＜ 0.01, ^***^ *P* ＜ 0.001.

Previously, it has been demonstrated that FOXP4 mediates radioresistance in CRC by regulating ferroptosis. Therefore, we explored the mechanism by which FOXP4 regulates ferroptosis. Considering that FOXP4 is a transcription factor of the FOXP family, it regulates the activation and expression of target genes via its FH structural domain. Accordingly, we knocked down FOXP4 and analyzed its downstream target genes using the Cut&Tag assay. Based on the analysis, GPX4 was identified as the downstream target gene (**Figure** **6**A; Table S11–12, Supporting information). Additionally, we designed a FOXP4 plasmid to knockout the FH structural domain (FOXP4^FH‐Del^). Moreover, the promoter region of GPX4, which is transcriptionally modified by FOXP4, was predicted according to the JASPAR dataset, and the sequence was mutated to design a mutant plasmid (GPX4‐Mut‐Luc) (Figure 6B).

Luciferase assay showed that FOXP4 suppression downregulated its interaction with the promoter region of GPX4, whereas FOXP4 overexpression enhanced both interactions. In contrast, FOXP4 overexpression failed to increase its interaction with the mutated GPX4 (partial sequence of the promoter region was mutated) (Figure 6C). Collectively, these data indicate that FOXP4 functions in the promoter region of GPX4 and its interaction sequence is “AGGTAAATAAG”. Additionally, chromatin immunoprecipitation (ChIP) and PCR showed that suppressing FOXP4 expression significantly decreased the amount of DNA fragments of GPX4 (Figure 6D,E). Moreover, FOXP4 upregulation enhanced its interaction with GPX4 promoter region. However, overexpression of FOXP4 mutant lacking the FH domain did not significantly upregulate GPX4 transcription (Figure 6F; Figure S6A, Supporting Information). Furthermore, ChIP‐PCR confirmed that the transcription factor FOXP4 interacted with the promoter region of GPX4 and that this interaction was dependent on the FH structural domain of FOXP4. Then, we verified the effects of FOXP4 expression on GPX4 transcription and protein levels at different time points after irradiation to further analyze the regulatory mechanisms between FOXP4 and GPX4 under irradiation. Our results showed that the protein expression of FOXP4 gradually increased at 0,4,8,12,24, and 48 h after irradiation in HCT116 and HCT15 cells, whereas the protein expression of GPX4 showed a tendency of decreasing and then increasing (Figure S5A, Supporting Information). And in our previous results of hepatocellular carcinoma cells undergoing ferroptosis, we also obtained a similar trend for GPX4.^[^ ^5^ ^]^ Combining the trends of the two papers, we inferred that GPX4, as the peroxidase, could reduce the lipid peroxides produced by irradiated to alleviate the oxidative damage of tumor cells. Therefore, GPX4 at 4–8h after irradiation was reduced due to its involvement in the redoxidation reaction of lipid peroxides. Subsequently, the content of GPX4 was gradually regained due to the initiation of the transcription process. We then detected the transcript levels of GPX4 after irradiation by Luciferase assay and PCR assay (Figure S5B,C, Supporting Information). The results showed that in HCT116 cells, the binding of FOXP4 and GPX4 promoter region was elevated at 4,8,12,24, and 48h of irradiation compared to 0h after irradiation, with the highest value at 12h. Similarly, mRNA expression of GPX4 was elevated at 4,8,12,24, and 48h compared to 0h after irradiation. In HCT15 cells, the binding of FOXP4 to the GPX4 promoter region was also elevated at other time points within 48h of irradiation compared to 0 h after irradiation, as was GPX4‐mRNA expression. This indicated that the elevated transcript levels of GPX4 directed by FOXP4 indeed compensated for the irradiation‐induced decrease in GPX4 protein levels.

Notably, previous studies have shown that FOXP1, FOXP2 and FOXP4 tend to rely on their leucine zip (LZ) motif interactions to exert transcriptional modifications.^[^ ^18^ ^]^ Specifically, they worked through LZ motifs to foster autodimerization or heterodimerization into dimers with each other to bind DNA sequences of target genes and facilitate transcriptional modification. Therefore, we analyzed the protein structural domains of FOXP1, FOXP2, and FOXP4 (Figure 6G). We predicted structural diagrams for the formation of dimers between FOXP4 and itself, and with FOXP1 or FOXP4 based on Alpha fold2 (Figure 6H). Co‐IP assay confirmed that FOXP4 can interact with FOXP1 and FOXP2 via the LZ motif (Figure 6I). Additionally, we observed that the heterodimerization of FOXP4 with FOXP1 and FOXP2 contributed considerably to the transcriptional modification of GPX4 compared with autodimerization. Importantly, the transcriptional efficiency of FOXP4 on GPX4 was greatly reduced after mutating the LZ motifs (Figure 6J). Collectively, these findings indicate that FOXP4 can heterodimerize with FOXP1 and FOXP2 or autodimerize with itself through the LZ motif and promote the transcriptional modification of the promoter region (AGGTAAATAAG) of GPX4 and ultimately regulate the onset of ferroptosis in CRC.

### 2.7 Doxorubicin Improves Radiotherapy by Targeting FOXP4

Research findings indicate that FOXP4 inhibits ferroptosis and promotes radioresistance by regulating the transcriptional modification of GPX4 in CRC. Therefore, identifying potential drugs targeting FOXP4 to enhance the efficacy of radiotherapy in CRC is promising. Accordingly, we screened drugs that interact and inhibit FOXP4 expression, including valproic acid, doxorubicin (DOX), resveratrol, and afimoxif, using the PubChem website (Figure S6B, Supporting Information). DOX is a broad‐spectrum anthracycline‐based chemotherapeutic agent; therefore, we assumed that prioritizing the sensitizing effect of DOX in radiotherapy targeting FOXP4 would have better clinical research prospects.

**Figure 7:**
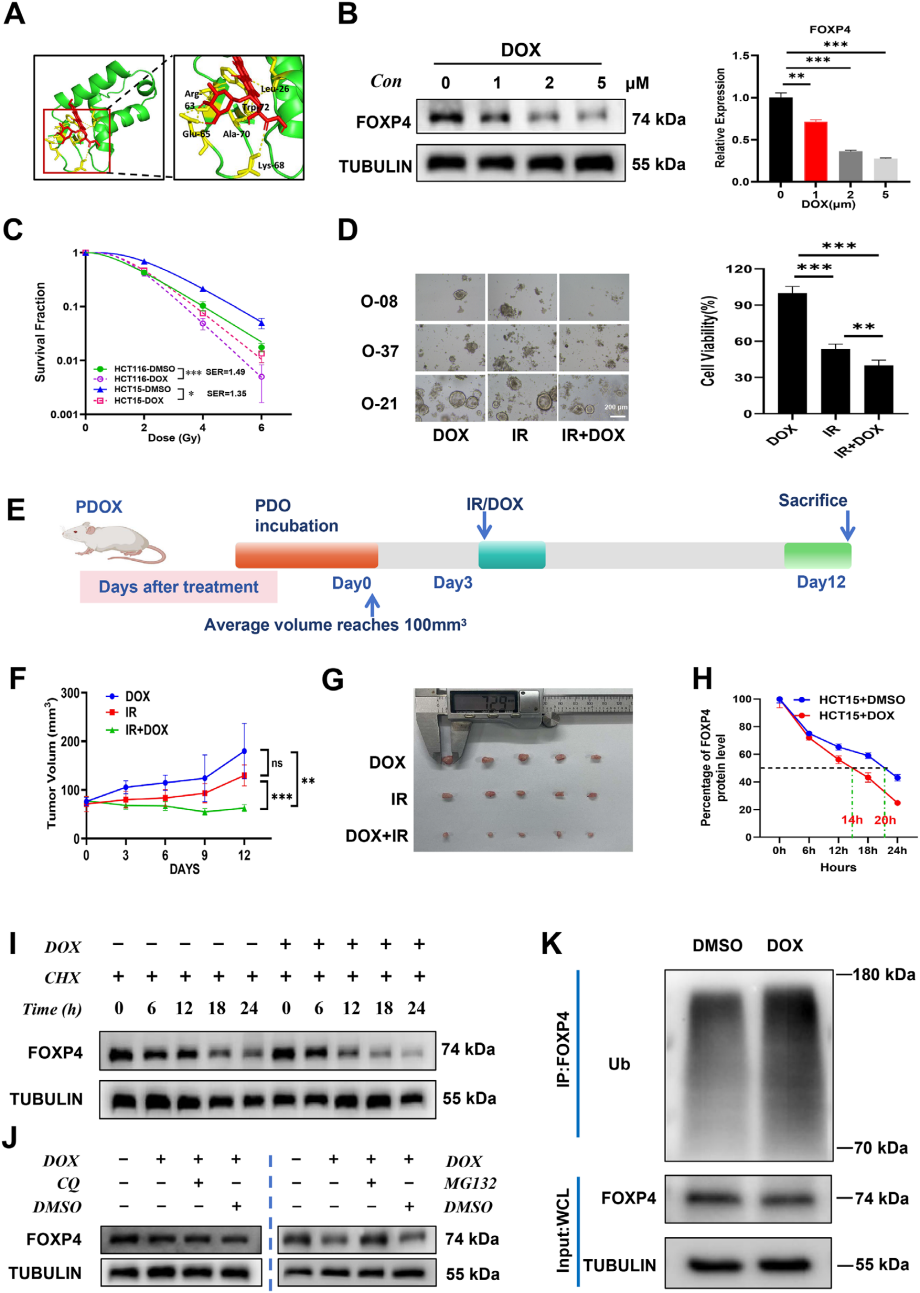
DOX enhanced radiosensitivity of CRC by promoting the ubiquitination of FOXP4. A) Cartoon illustration showed the interaction between DOX and FOXP4. B) Representative images and quantification of western blot assay of FOXP4 and α‐tubulin proteins in HCT15 cells treated with DOX. C) Dose responses of survival factions of HCT116 and HCT15 cells treated with or without DOX. D. Bright field image and quantitative results of PDOs treated with DOX after 8Gy irradiation. Scale bar, 200 µm. E) Pattern plots of PDOX treated with DOX that were irradiated at 8Gy*3d and then executed at the appropriate time. F) PDOX tumor volume of DOX, IR and DOX+IR groups was examined every 3 days until 9 days after IR(8Gy*3d). G) General view of tumor mass of each indicated group at 9 days after IR(8Gy*3d). H,I) Point‐fold line chart (H) of FOXP4 protein degradation according to western blot assay (I) showed the expression of FOXP4 protein at different time points after 4 Gy IR in HCT15 cells with or without DOX after CHX treatment. J) Western blot analysis of FOXP4 and α‐tubulin proteins in HCT15 cells at 4 h after 4 Gy IR. MG‐132 (10µM) or CQ (50µM) was added before IR. K) Anti‐Ub immunoblotting assay of FOXP4 polyubiquitination in HCT15 cells at 4h after 4Gy. ^*^ *P* ＜ 0.05, ^**^ *P* ＜ 0.01, ^***^ *P* ＜ 0.001.

To identify the possible combined action of FOXP4 and DOX, we performed molecular docking using AutoDock software and found that the docking binding energy was ˗5.84 kcal/mol, suggesting that docking was possible (**Figure** **7**A). Additionally, we examined the effect of DOX treatment on FOXP4 protein expression using western blotting and found that DOX decreased FOXP4 expression in a dose‐dependent manner (Figure 7B). Moreover, we investigated the effect of the DOX‐induced decrease in FOXP4 expression on CRC radiosensitivity at the cellular, organoid, and animal levels. At the cellular level, treatment with 1 µm of DOX (half‐maximal inhibitory concentration [IC_50_], 2.989, Figure S6C, Supporting Information) significantly reduced the clonogenic rate of HCT15 and HCT116 cells after irradiation compared with those in the control group (Figure 7C). At the PDOs level, DOX markedly inhibited the growth of PDOs after irradiation compared with that in the control group (Figure 7D). In vivo, DOX‐treated nude mice transplanted with PDOX and HCT15 cells showed slower tumor growth and reduced tumor size after irradiation at 8 Gy for 3 days (Figure 7E–G; Figure S6D–F, Supporting Information). Collectively, these results indicate that drugs targeting FOXP4 increase CRC radiosensitivity at the cellular, organoid, and animal levels.

To elucidate the underlying mechanism of DOX, we examined the variations in FOXP4 transcription and protein levels after DOX treatment. Although DOX had no effect on FOXP4 transcription (Figure S6G, Supporting Information), it impaired FOXP4 protein degradation, decreasing its half‐life from 20 to 14 h (Figure 7H,I). To clarify the mechanism of DOX‐induced FOXP4 protein degradation, HCT15 cells were cotreated with DOX and chloroquine (CQ) or MG132, and changes in FOXP4 protein degradation were observed. DOX‐induced degradation of FOXP4 protein was blocked by MG132 but not by CQ, indicating that DOX tended to promote FOXP4 protein degradation via the ubiquitin‐proteasome pathway rather than through the autophagic lysosomal pathway (Figure 7J; Figure S6H, Supporting Information). To further verify this ubiquitination function, we performed Co‐IP assay on whole‐cell proteins treated with DOX and found that FOXP4 ubiquitination level was higher in the DOX group than in the control group (Figure 7K; Figure S6I, Supporting Information). Collectively, these findings indicate that DOX promotes FOXP4 protein degradation through the ubiquitin‐proteasome pathway to increase CRC radiosensitivity, providing a new clinical option for the radiotherapy of patients with CRC with high FOXP4 expression.

Overall, high FOXP4 expression promotes GPX4 transcription, which inhibits ferroptosis and induces radioresistance in CRC. In contrast, DOX induces FOXP4 downregulation through ubiquitination degradation, thus inducing radiosensitization in CRC with high FOXP4 expression.

## 3 Discussion

Recently, several basic and clinical studies have been conducted by national and international research teams to improve the CR rate of neoadjuvant therapy in patients with CRC (especially rectal cancer). The TORCH and UNION studies improved the CR rate of patients with LARC to a maximum of 56.5% by optimizing the treatment modality and combining short‐course radiotherapy with systemic therapy.^[^ ^19^ ^]^ The REGINA study increased the CR rate to 45.8% by combining targeted therapy with induction chemotherapy, followed by sequential short‐course radiotherapy to activate the immune system. In another clinical study, neoadjuvant chemotherapy and long‐range radiotherapy were combined with PD‐1 inhibitors to treat patients with pMMR rectal cancer, and a final CR rate of 44.8% was achieved.^[^ ^20^ ^]^ Combining chemotherapy, targeted therapy, or immunotherapy with radiotherapy could enhance the CR rate of patients with rectal cancer. Therefore, identifying the mechanism underlying radioresistance and screening for potential therapeutic targets will contribute to improving the efficacy of neoadjuvant radiotherapy, ultimately increasing the CR rate and improving the quality of life of patients with rectal cancer.

In the present study, we identified ferroptosis as a key mechanism mediating radioresistance in CRC based on transcriptomic analysis of cell lines and PDOs. Ferroptosis, as an iron‐dependent and lipid peroxide‐driven programmed cell death, is closely related to tumor development, progression, and treatment.^[^ ^7^, ^9^, ^10^ ^]^ Considering that Fe^2+^ mainly accumulates in organs such as the liver and spleen and is reabsorbed in the intestine to maintain systemic iron homeostasis,^[^ ^21^ ^]^ investigating the role of ferroptosis in CRC radioresistance will enhance our understanding of systemic iron homeostasis. Importantly, several studies have confirmed that ferroptosis is correlated with the efficacy and prognosis of tumor radiotherapy.^[^ ^22^ ^]^ Although radiotherapy elevates lipid peroxides to induce ferroptosis, it simultaneously activates anti‐ferroptosis pathways, contributing to radioresistance.^[^ ^22^, ^23^ ^]^ Our findings confirmed that ferroptosis was one of the “killers” inducing cell death after radiotherapy. Insufficient ferroptosis activation is the main mechanism underlying radioresistance development. Although GPX4, a hallmark negative regulator of ferroptosis, was highly expressed in both radioresistant cells and organoids, its differential expression between CRC and normal tissues was not significant. Overall, these results indicate that GPX4 has poor diagnostic significance and targetability in clinical settings. Therefore, screening for other genes that regulate ferroptosis and are differentially expressed in CRC is of great clinical value.

Our in vivo and in vitro experiments indicated that FOXP4 inhibition markedly increased CRC radiosensitivity. FOXP4 functions as a transcription factor that modulates gene expression alone, or via co‐activator/repressor complexes with FOXP1/2.^[^ ^11^, ^24^ ^]^ In the present study, FOXP4 bound FOXP1/2 as heterodimers or form homodimers with itself via its LZ motifs, jointly promoting GPX4 transcription and inhibiting ferroptosis. FOXP4 contributes to the transcriptional modification of GPX4 via its FH structural domain. Although ferroptosis inducers, such as erastin and RSL3, target GPX4 to trigger cell death, their clinical application is limited by poor water solubility, unstable metabolism, and low selectivity.^[^ ^9^, ^25^ ^]^ Therefore, it would be of clinical significance to antagonize GPX4 transcription via FOXP4 inhibition.

Research findings indicate that erastic‐ and irradiation‐induced ferroptosis activation has different regulatory effects on certain pathways and marker proteins.^[^ ^7^, ^8^ ^]^ Compared with erastin treatment, irradiation‐induced ferroptosis activation was more likely to lead to high FOXP4 protein expression, thereby limiting further increasing in ferroptosis. However, DOX treatment combined with irradiation may attenuate the compensatory increase in FOXP4 expression, thereby further increasing irradiation efficacy in CRC cells. As a broad‐spectrum anthracycline antitumor agent, DOX induces DNA damage and promotes free radical formation by inhibiting topoisomerase II.^[^ ^26^ ^]^ Irradiation can also generate ROS through the ionization of water molecules or directly target the DNA of tumor cells and trigger DNA double‐strand breaks to attack tumor cells. In our study, DOX treatment effectively inhibited FOXP4 expression via ubiquitination. Therefore, DOX treatment combined with radiotherapy may not only increase the conventional antitumor efficacy but also eliminate the radioresistance caused by high expression of FOXP4 in CRC, thereby enhancing sensitization to radiotherapy. DOX‐induced ubiquitination of target proteins has been previously described. For example, SIRT1 attenuates DOX‐induced cardiotoxicity by disrupting the SESN2–MDM2 interaction, thereby mitigating DOX‐induced SESN2 ubiquitination. *Momordica charantia* L‐derived exosome‐like nanovesicles (MC‐ELNs) stabilized p62 protein levels by suppressing DOX‐induced degradation of p62 ubiquitination, which promoted the expression of HO‐1 and ultimately facilitated the survival of cardiomyocytes.^[^ ^27^ ^]^ Mechanistically, DOX‐induced cardiotoxicity is attributed to impaired DNA/RNA synthesis, oxidative stress, Ca^2^⁺ dysregulation, and endoplasmic reticulum expansion in cardiomyocytes.^[^ ^28^ ^]^ Notably, the angiotensin‐converting enzyme inhibitors (ACEIs), metformin, and idebenone, effectively reduced the cardiotoxicity of DOX.^[^ ^29^ ^]^ Furthermore, liposomal DOX has been shown to significantly attenuate cardiotoxicity and enhance its antitumor efficacy when coated with polyethylene glycol.^[^ ^30^ ^]^ Accordingly, the simultaneous application of liposomal DOX and radiotherapy or the combination of DOX and cardioprotective agents (such as metformin) with simultaneous radiotherapy offers a promising therapeutic strategy for CRC.

Conclusively, we identified FOXP4 as a potential radioresistance gene that induces radioresistance by suppressing ferroptosis activation in CRC. Mechanistically, FOXP4 forms a heterodimer with FOXP1/2 or a homodimer through the LZ motif and regulates GPX4 transcription by binding to the AGGTAAATAAG sequence in the promoter region of GPX4 via the FH domain. Additionally, DOX promotes ferroptosis after radiotherapy by targeting FOXP4 ubiquitination and degradation. Consequently, the DOX‐radiotherapy combination modality provides a new strategy for enhancing therapeutic efficacy in patients with CRC. Additionally radiotherapy, particularly hypofractionated radiotherapy, can induce immunogenic cell death and promote antigen presentation, thereby enhancing the efficacy of immunotherapy. While inhibition of FOXP4 may further increase tumor radiosensitivity by inducing ferroptosis. Therefore, combining FOXP4‐targeted therapies (such as doxorubicin) with radiotherapy, followed by sequential immunotherapy, may not only overcome radioresistance but also enhance the efficacy of antitumor immunotherapy. This therapeutic strategy is currently being investigated in clinical studies conducted by our team.

## 4 Experimental Section

Patients with rectal cancer (*n* = 96) receiving neoadjuvant radiotherapy were recruited in Zhejiang Provincial Cancer Hospital. All patients received 45–50.4 Gy/25‐28F radiotherapy with simultaneous capecitabine/ 5‐Fu‐based chemotherapy. WES, RNA sequencing, H&E staining, and immunohistochemistry (IHC) assay were performed on tumor tissues and PDOs (Tables S1–S8, Supporting Information). Cell lines, organoids, and xenograft mouse models were used to investigate the function of FOXP4. WB, RT‐qPCR, Cut&Tag sequencing, irradiation assays, clonogenic assay, CCK‐8 assay, ROS measurement, MDA assay, lipid peroxide measurement, Co‐IP assay, ubiquitylation assay, dual‐luciferase reporter assay, Chromatin Immunoprecipitation (ChIP) assay, and EMSA were conducted to explore the roles and mechanisms of FOXP4 in radioresistance (Tables S9–S12, Supporting Information). A detailed description of the materials and methods is provided in the Materials and Methods (Supporting Information).

### Ethics Statement

The acquisition and subsequent utilization of clinical samples were approved by the Ethics Review Committee of Zhejiang Cancer Hospital (Hangzhou, China), and informed consent was obtained from patients or their family members (IRB‐2021‐291; IRB‐2022‐677; IRB‐2018‐58). The animal study protocol was approved by the Animal Welfare and Ethics Committee of Zhejiang Cancer Hospital (2024‐02‐016).

## Conflict of Interest

The authors declare no conflict of interest.

## Author Contributions

Q.C., Y.J., L.L., R.S. contributed equally to this work. Q.C., X.L., W.M. and J.Z. manuscript writing, conceptualization, data analysis and Funding acquisition. Y.J., L.L., R.S., and X.G. experimental manipulation. X.G., Q.J., and X.T. animal experiments manipulation and clinical sample collection & analysis. Q.D. and Y.H. Bioinformatics analysis. Q.S., D.L., L.L., T.L., and Z.H. clinical patient enrollment, clinical information collection, MRI, IHC & IF results determination and Funding acquisition.

## Supporting information



Supporting Information

Supporting Information

Supporting Information

Supporting Information

Supporting Information

Supporting Information

Supporting Information

Supporting Information

Supporting Information

Supporting Information

Supporting Information

Supporting Information

Supporting Information

Supporting Information

Supporting Information

## Data Availability

The data that support the findings of this study are available from the corresponding author upon reasonable request.
