# Supplementary material for: Patients‐Derived Organoids Sequencing‐based FOXP4 Facilitates Radioresistance by Transcriptionally Modifying GPX4 to Regulate ferroptosis in Colorectal Cancer
Source: Adv Sci (Weinh). 2025 Aug 11;12(37):e07080. doi: 10.1002/advs.202507080 (PMC12499432; doi:10.1002/advs.202507080)
Supplement: Supplementary file 1 — Supporting Information [file ADVS-12-e07080-s010.pdf]

ADVANCED SCIENCE
Open Access

Supporting Information

for *Adv. Sci.*, DOI 10.1002/advs.202507080

Patients-Derived Organoids Sequencing-based FOXP4 Facilitates Radioresistance by Transcriptionally Modifying GPX4 to Regulate ferroptosis in Colorectal Cancer

Qianping Chen, Yuzhao Jin, Luyu Liao, Rong Shen, Xingnan Ge, Qingyu Jiang, Ying Huang, Quanquan Sun, Dong Liu, Luying Liu, Tongxin Liu, Qinghui Dai, Xiyuan Tang, Zhe Han, Xin Gao, Xinhua Lin*, Wei Mao* and Ji Zhu**

Supplementary Materials and Methods

Patient samples

96 rectal cancer patients receiving neoadjuvant radiotherapy were from Zhejiang Provincial Cancer Hospital. All of them received 45-50.4Gy/25-28F radiotherapy with simultaneous capecitabine/ 5-Fu- based chemotherapy. They underwent MR examination before and after radiotherapy to assess tumor regression after neoadjuvant radiotherapy. Clinical information was provided in Supplementary Table S1.

The mrTRG scores were assessed by specialist radiologists based on the MERCURY experience, according to the MRIs of the patients before and after neoadjuvant radiotherapy. The mrTRG score ranged from 1 to 5, with mrTRG 1 defined as almost no tumor signal present. mrTRG 2 was defined as more than 75% fibrotic signal with minimal tumor remnants. mrTRG 3 was defined as more than 50% fibrotic signal with visible tumor remnants. mrTRG 4 was defined as a majority of tumor signal, with minimal fibrotic signal intensity. mrTRG 5 was defined as almost no change from the primary tumor and little or no visible fibrotic signal. Due to the presence of radiological enteritis after radiotherapy, inflammatory and tumor signals can interfere with each other, and 50% fibrosis signal judgement may be inaccurate, so we did not classify patients with mrTRG score 3. Instead, patients with mrTRG scores 1-2 were defined as radiosensitive patients, and mrTRG 4-5 were defined as radioresistant patients.

Most of the 96 patients were included in the clinical study (ClinicalTrials. gov NCT05669092). Additionally, clinical colorectal cancer samples and paracancerous tissues were obtained with the consent of the biospecimen repository and with ethical approval from the Zhejiang Cancer Hospital. (Ethics No.: IRB-2021-291; IRB-2018-58; IRB-2022-677).

Organoids tissue acquisition

Supported by the ethical approvals mentioned above, we performed pre-treatment anoscopic biopsies of patients undergoing neoadjuvant radiotherapy. Tumor tissue (volume ＞15 mm^3) obtained from the biopsy was divided into four pieces, one for pathology and immunohistochemistry, one for organoid construction, and two for WES sequencing and RNA-seq sequencing, respectively.

The experiments using human organoids in this study were approved by the Ethical Review Committee of Zhejiang Cancer Hospital (Hangzhou, China), approval number: IRB-2021-291.

PDOs irradiation assay and drug tests assay

CRC organoids culture and organoids preparation for irradiation response assays and drug tests assays was performed as previously described (***PMID: 31761724***). For irradiation tests, organoids were exposed to X-rays of 8Gy. Next, we matched PDOs outcomes after irradiation with patients' clinical responses after radiotherapy to screen 20 radiosensitive and 20 radioresistant organoids, as detailed in

Supplementary Table S2.

For drug tests assay, organoids were treated with 10 μM Erastin or 10 μM DOX with or without 8Gy IR. After 24 days, cell titer assay was performed to evaluated organoids cell viability according to manufacturer's instruction (Promega, Wisconsin, USA)

Cell culture

Human CRC cell line HCT15 was purchased from the ATCC Cell Bank (Manassas, VA, USA). HEK293 cells and human CRC cell line HCT116 were purchased from Shanghai Cell Bank (Chinese Academy of Science, Shanghai, China). Radioresistant cell line HCT116R and HCT15R were constructed by HCT116 and HCT15 cells receiving 25 × 2Gy at a dose rate of 1Gy/min. Cells were cultured in DMEM high-glucose medium (containing 10% fetal calf serum, 1% streptomycin and penicillin) in a 5% CO_2, 37°C incubator. All cell lines were screened for mycoplasma by the investigator every 2 months during the culture process to ensure the absence of mycoplasma infection. All cell lines were identified by short tandem repeat (STR) assay. The STR identification of HEK293 was Amelogenin: X, X, D5S818: 8, 9, D13S317: 12, 14, D7S820: 11, 11, D16S539: 9, 13, vWA: 16, 19, TH01: 7, 9.3, TPOX: 11, 11, CSF1PO: 7, 12, D19S433: 15, 18, D21S11: 28, 30.2, D18S51: 17, 18. The STR identification of HCT116 cells was Amelogenin: X,Y, D5S818: 10,11, D13S317: 10,12, D7S820: 11,12, D16S539: 11,13, vWA: 17,22, TH01: 8,9, TPOX : 8,9, CSF1PO: 7,10, D19S433: 12,13, D21S11: 29,30, D18S51: 17. The STR identification of HCT15 cells was: Amelogenin: X,Y, CSF1PO: 12, D13S317: 8,11, D16S539: 12,13, D5S818: 13, D7S820: 10,12, TH01: 7,9.3, TPOX: 8,11, vWA: 18,19, D3S1358: 17, D21S11: 29,32.2, D18S51: 11,17, D8S1179: 15, D19S433: 14,16, D2S1338: 17,25.

Plasmid construction, lentivirus packaging and infection

Using full-length FOXP4 amplicons as templates, the FH domain deletion plasmid ($FOXP4^{FH\text{-}Del}$) and the LZ domain mutation plasmid ($FOXP4^{LZ\text{-}Mut}$) were amplified by PCR and cloned into Flag-tagged pcDNA3.1 vectors (GenePharma, Shanghai, China). Non-targeting shRNA sequences were used as negative control (shNC) and shRNA targeting FOXP4 (shFOXP4) was used as experimental group. Non-targeting sgRNA sequences were used as negative control (sgNC) and sgRNA targeting FOXP4 (sgFOXP4) was used as experimental group. Plasmid of shNC, shFOXP4, sgNC and sgFOXP4 were constructed and provided by Hanyin Biotechnology Co. The shFOXP4 sequence was TTCGCCTATTTCCGCAGAA and the sgFOXP4 sequence was GAGCACAGTAGGCTGTCCGT. The promoter sequences of wild-type (which was called GPX4-Luc: -1899bp to 100bp, containing DNA binding region -1537 to -1547) and mutant types (which was called GPX4-Mut- Luc: mutated -1537 to -1547 AGGTAAATAAG to TCCATTTATTC) of GPX4 were cloned into the pGPL4 plasmid (GenePharma, Shanghai, China).

For lentivirus packing, HEK293 cells were transfected with a mixture of the plasmid mentioned above, psPAX2 packaging plasmid, pMD2.G envelope plasmid, OPTI-MEM and FuGENE reagent (Promega, USA). Culture medium was collected 48h after transfection and stored at −80 °C.

HCT15 and HCT116 cell lines were infected with lentivirus of shNC, shFOXP4, FOXP4, FOXP4^{FH-Del} and FOXP4^{LZ-Mut}. The antibiotic-resistant transfected cells were selected and enriched by treatment of puromycin (Hanyin Biotechnology Co., Shanghai, China). The efficiency in CRC cells and organoids was monitored by Western blot assay.

Sequencing assays

DNA extractions and Whole exome sequencing

500ng DNA of organoids and tumor tissues from CRC patients was extracted using aDNeasy Blood & Tissue Kit (Qiagen, Hilden, Germany). For each sample, DNA was sheared and subject to whole exome sequencing using the Agilent v2 capture probe set and sequenced by HiSeq2500 and 150bp paired-end reads were generated.

RNA extractions and total RNA sequencing

Total RNA of organoids and tumor tissues from CRC patients and cell lines (HCT116, HCT116R, HCT15 and HCT15R) was extracted using the RNeasy Mini Kit with on-column DNase digestion (Qiagen, Hilden, Germany). Paired-end libraries were prepared using the ABclonal mRNA-seq Lib Prep Kit (ABclonal, China) following the manufacturer's instructions, then sequencing was performed with an Illumina Novaseq 6000 instrument. Raw data was quality checked using Fastqc and transcripts were aligned to reference genome hg19 using HISAT2 and quantified by DESeq2.

Cut&Tag assay and sequencing

The Cut & Tag assay was carried out utilizing the NovoNGS® CUT & Tag 4.0 High-Sensitivity Kit following the manufacturer's protocols (Novoprotein, Suzhou, China). Briefly, HCT15-shNC, HCT15-shFOXP4, HCT116-shNC and HCT116-shFOXP4 cells were incubated with activated NovoNGS ConA Beads. Subsequently, the bead-bound cells were permeabilized and incubated initially with FOXP4 primary antibody (Proteintech, Shanghai, China) as the primary antibody and a goat anti- rabbit IgG (Abcam, MA, USA) as the secondary antibody, a negative control group was included without the primary antibody. The diluted pAG-Tn5 adapter complex was then added, followed by the tagmentation reaction. The extracted DNA fragments were used for library preparation. The libraries were sequenced by Novogene on the Illumina NovaSeq 6000 platform. Sequencing data was detailed in Supplementary Table S11-12.

Databases description

GEPIA 2 database analysis

GEPIA 2 (http://gepia2.cancer-pku.cn, accessed 1st Jan, 2025) offered an enhanced and more comprehensive gene expression analysis tool based on data from the Cancer Genome Atlas (TCGA) and Genotype-Tissue Expression (GTEx) projects. GEPIA 2 included data from over 11,000 tumor samples and 9,000 normal samples, providing deeper insights into the expression profiles of over 20,000 gene across 33 cancer types.

In this study, GEPIA 2 was employed to explore the expression of FOXP4 in CRC and normal tissues. Additionally, survival analysis was conducted using log-rank and Mantel-Cox tests to evaluate the correlation between FOXP4 expression levels and DFS in CRC patients.

KMPlot database analysis

KMPlot (http://kmplot.com/analysis, accessed 1st Jan, 2025) was a web-based platform for survival analysis based on gene expression data from multiple cancer types. It integrates data from several major public datasets, including TCGA, GEO and the European Bioinformatics Institute, offering survival data for over 54,000 samples from 21 cancer types.

In this study, KMPlot was utilized to analyze the correlation between FOXP4 expression and recurrence free survival in CRC patients.

PubChem database analysis

PubChem (https://pubchem.ncbi.nlm.nih.gov, accessed 1st Jan, 2025) was a comprehensive public chemical database maintained by the National Center for Biotechnology Information (NCBI). It provided detailed information on the biological activities of small molecules, including their chemical structure, properties, and interactions with various biological targets. In this study, PubChem was used to search for chemical compounds that interact with the FOXP4 gene which were potential as therapeutic agents for targeting FOXP4 in CRC.

Bioinformatics analysis

All dataset differential analyzes were performed by the limma package. 5063 differential genes were obtained from the TCGA data between CRC tissues and normal tissues set with fold change (FC)$\geq$2.0 or $\leq$0.5 and p-value $<$ 0.01. 212 differential genes were obtained from the organoid sequencing data between radioresistant and radiosensitive PDOs set with FC$\geq$1.5 or $\leq$0.667 and p-value $<$ 0.01. 8794 differential genes were obtained from the HCT116R/HCT116 dataset with FC$\geq$1.5 or $\leq$0.667 and p- value <0.01; 8400 differential genes were obtained from the HCT15R/HCT15 dataset with FC$\geq$1.5 or $\leq$0.667 and p-value <0.01; 7766 differential genes were obtained from the HCT15/HCT116 dataset with FC$\geq$1.5 or $\leq$0.667 and p-value <0.01. The information was detailed in Supplementary Table S4-S8.

The differential gene enrichment pathways between resistant cell lines and sensitive cell lines were derived from KEGG (https://www.genome.jp/kegg). The relationship between gene expression was determined by the spearman method.

Irradiation assay

The irradiation instrument was an X-ray bio-irradiator (SARRP3, X trahl, USA). The irradiation dose rate was 1Gy/min for cells and PDOs, and 2Gy/min for mice. The irradiation dose rates for cells were 2,4, 6 and 8Gy, for PDOs was 8Gy, and for mice was 8Gy* 3 days. Irradiation time varied with the dose setting. In the *in vivo* experiments, mice bearing CRC transplant tumors were anaesthetised withketamine/chlorpromazine (100 mg/kg + 10 mg/kg) prior to irradiation, followed by irradiation of themouse tumors.

Clonogenic assay

Colony formation assay was used to determine the radiosensitivity of CRC cells. CRC cells (150-1200 cells/well) were seeded into 6-well plates and treated with various doses of IR (0, 2, 4, 6Gy) in triplicate after cell attachment on the following day. Colonies were formed 9-12 days post-IR, then methanol-fixed and stained with crystal violet. Cell survival curves after IR were fitted based on the single-click multi-target model (Equation: SF = 1-(1-exp(-k*D))^N).

CCK-8 Assay

CCK-8 assays were performed according to the protocol provided by the manufacturer (Beyotime Biotechnology, Shanghai, China). In summary, 5000 cells (100µL of culture medium) were placed in each well of a 96-well plate and treated with drug or irradiation after 24h. After 48h of treatment, 10 µL of CCK-8 solution was added to each well of the plate, incubated for 1h and measured the absorbance at 450nm using a microplate reader (Thermo Scientific™, MA, USA).

ROS Measurement

After 6h of irradiation or drug treatment, HCT116 and HCT15 cells were added with a 1:1000 dilution of the DCFH-DA probe (Beyotime Biotechnology, Shanghai, China), and incubated at 37°C for 30 minutes. After removing the supernatant, cells were washed three times with PBS, and took pictures under a high-content imaging system (MD ImageXpress Micro XL, Molecular Devices, CA, USA). Fluorescence intensity was measured and analyzed using image J (version no.:1.8.0_345; https://imagej.nih.gov/ij/; NIH, USA).

Lipid peroxides Measurement

After 6h of irradiation or drug treatment, 10 µM liperfluo (Dojingo Molecular Technologies Inc., Shanghai, China) was added to HCT116 and HCT15 cells and incubated at 37°C for 30 minutes. After removing the working solution, cells were washed three times with PBS, and took pictures under a high-content imaging system (MD ImageXpress Micro XL, Molecular Devices, CA, USA). Fluorescence intensity was measured and analyzed using image J (NIH, USA).

MDA assay

100,000 cells were cultured overnight in 6-well plates. After 6h of drug or irradiation treatment, we performed the MDA assay according to the instructions of the MDAAssay Kit (Dojingo, Molecular

Technologies Inc., Shanghai, China). The obtained samples were measured with a microplate reader (Thermo Scientific™, MA, USA) to detect fluorescence intensity (Ex: 540 nm, Em: 590 nm), and then the MDA concentration in the sample was calculated through the MDA standard curve.

Xenograft tumor mouse model

Four-week-old female nude mice were ordered from SLAC Experimental Animal Co., Ltd., Shanghai. The cells were randomly divided into cages and cultured for two weeks. After adapting to the environment, tumor cells were inoculated. Prepare $1*10^6$/100μL cell suspension, inject 100μL cell suspension subcutaneously into the left abdomen of nude mice, and observe whether tumors form after one week. When the tumor reached an average size of $100mm^3$, irradiation treatment and intraperitoneal injection of drugs were performed.

Four-week-old female NSG rats were ordered from Model Organisms Center, LNC, Shanghai. They were randomly divided into cages and cultured for two weeks. After adapting to the environment, they were inoculated with biopsy tumor tissue from clinical patients. The tumor tissue was cut to a suitable size, and the tumor tissue was placed under the skin of the left abdomen of the NSG mouse with a puncture needle. After one week, it was observed whether tumors had formed. When the tumor reached an average size of 80 mm^3, irradiation treatment and intraperitoneal injection of drugs were performed.

Measured the tumor every three days after treatment. The calculation method of tumor volume was: $V=L\times W^2/2$ (L=length, W=width). When the maximum tumor volume almost reached $1000mm^3$, all grouped mice were killed and photographed.

For nude mice and NSG mice experiments, all investigations complied with the *ARRIVE* (Animal Research: Reporting of Experiments *in vivo*) guidelines and were performed in accordance with the NIH Guide for the Care and Use of Laboratory Animals (NIH Publication No. 8023, revised 1978). The animal experiment protocol was approved by the Animal Welfare and Ethics Committee of Zhejiang Cancer Hospital (2024-02-016).

H&E and IHC assay

The samples included tumor tissues and paracancerous normal tissues from CRC patients, PDOs samples and subcutaneous transplantation tumor of mice. The samples were fixed informalin, dehydrated in 75% ethanol, embedded in paraffin and cut into 4μm thick paraffin sections. For H&E staining, paraffin-embedded sections were stained with hematoxylin and eosin (H&E), then observed and photographed under a microscope. For IHC, tumor blocks were stained with antibodies against 4HNE, FOXP4, GPX4, Ki67, CDK2, CK20, CK-pan, β-catenin and counterstained with hematoxylin. The sections were then washed and incubated with appropriate secondary antibodies. Details of relevant antibodies are shown in Supplementary Table S9.

H&E and IHC images were obtained using a whole slide scanning system (Olympus VS200-BU, Japan) and analyzed semi-quantitatively using Image J software (NIH, USA). The index of analysis was the average optical density (AOD; AOD = Integrated optical density (IOD)/Area).

Western Blotting (WB) assay

Protein lysates (Beyotime Biotechnology) were added to the treated cells, and subsequently the lysates were subjected to protein quantification using aBCA quantification kit (Beyotime Biotechnology). 30μg of total protein was subsequently uploaded and separated on a 10% SDS-PAGE gel, transferred onto apolyvinylidene difluoride (PVDF) membrane (Immobilon-P; Millipore Co., MA, USA), and the PVDF membrane containing the proteins was later transferred to a containment solution and closed for 1h. The membranes were then incubated overnight at 4 °C with the corresponding primary antibody. On the next day, after three rinses with three-phase buffered saline containing 0.1% Tween 20, the membranes were incubated with horseradish peroxidase-conjugated IgG secondary antibodies (Biotech Biotechnology) for 1-2 h at room temperature. The bands were finally visualised using a chemiluminescence detection kit (Millipore, St. Louis, MO, USA). The detail information of relevant antibodies was listed in Supplementary Table S9.

Real-time quantitative PCR assay

Total RNA was extracted using an RNA extraction kit (DP451, TIANGEN, China), and complementary DNA (cDNA) was synthesized using HiScript III All-in-one RT kit (R333, Vazyme, China). Real-time PCR was performed using the SYBR Green kit (FP205, TIANGEN, China), and detection was performed by Real-Time PCR Instruments (ABI7500, Thermo Fisher Scientific, USA). 18S was used as control. For FOXP4, the forward primer was 5'-GGACACGGAGAGTGCAAGTG-3' and the reverse primer was 5'-GTGCTCTGTGTTGAGGTGTTT-3'. For GPX4 gene, the forward primer was 5'-GAGGCAAGACCGAAGTAAACTAC-3' and the reverse primer was 5'-CCGAACTGGTTACACGGGAA-3'.

Co-Immunoprecipitation (Co-IP)

Co-IP was performed according to the manufacturer's instruction. In brief, the whole cell lysates were collected and centrifuged at 10,000 g for 10 min at 4 °C. Then 1 mL supernatant was incubated overnight at 4 °C with 1μg anti-FOXP4 antibody or anti-IgG antibody and 20μL fresh protein A/G plus agarose beads (Santa Cruz Biotechnology, Shanghai, China). Samples were spun down, washed and fractionated by SDS-PAGE followed by Western blot analysis. Relevant antibodies were detailed in Supplementary Table S9.

Dual-luciferase reporter assay

HCT15-shNC, HCT15-shFOXP4, HCT116-shNC and HCT116-shFOXP4 cells were transfected with renilla luciferase vector and pGPL4-Basic or GPX4-WT-luc. Then HCT15-FOXP4^{WT}, HCT15-FOXP4$^{FH\text{-}Del}$, HCT116-FOXP4^{WT} and HCT116-FOXP4$^{FH\text{-}Del}$ cells were transfected with renilla

luciferase vector and pGPL4-Basic or GPX4-WT-luc or GPX4-Mut-luc. Cell lysates were collected for the luciferase assay 48h after transfection.

For time-course experiment, HCT15 and HCT116 cells were transfected with renilla luciferase vector and pGPL4-Basic or GPX4-WT-luc. After 24h transfection, cells were treated with 4Gy irradiation and Cell lysates were collected at 0, 4, 8, 12, 24, and 48h after irradiation for the luciferase assay.

Chromatin Immunoprecipitation (ChIP)

The ChIP assay was performed base on the manufacturer's protocol (Millipore) with a few modifications. Briefly, HCT15 and HCT116 cells were washed and collected after formaldehyde cross- linking. Then chromatin ware isolated andresuspended in ChIP lysis buffer containing protease inhibitor cocktail. Protein DNA complexes were immunoprecipitated and isolated DNA was analyzed by semiquantitative PCR using the following GPX4 promoter primer. the forward primer was 5'-ACAGCAAGACCCCGTGTCT-3' and the reverse primer was 5'-CTTAAGACTCGTCGAGGGGG-3'.

Electrophoretic mobility shift assay (EMSA)

His-FOXP1, His-FOXP2, His-FOXP4^{WT}, His-FOXP4$^{LZ\text{-}Mut}$ plasmids were constructed and protein purification was performed by BIORUN biosciences co. Wuhan, China. The 5'-biotinylated oligonucleotide (5'-AAAAAAGGTAAATAAGAGTTGGGGA-3') was used as the probes. The probes were incubated with the nuclear extract at room temperature for 30 min. The DNA binding activity was determined by a chemiluminescent EMSAKit (GS009, Beyotime, China) according to instruction. The entire reaction mixture was run on a non-denaturing 0.5×TBE 6% polyacrylamide gel and then transferred onto nylon membrane.

Drug treatment

CRC cells were treated with Erastin (10μM) or Fer-1 (5μM) for 6h to induce or inhibit ferropotosis, treated with 1μM of doxorubicin (DOX) for 12h to decrease FOXP4 for sensitizing radiotherapy, and treated with 10μM of MG132 for 4h to inhibit proteasome activity. Organoids were treated with 10μM of Erastin to induce ferropotosis, treated with 10μM of DOX to decrease FOXP4. In animal experiments, mice were pretreated with Erastin (10mg/kg) or DOX (2 mg/kg) for 24h and then received 8Gy x 3days. All of these drugs were purchased from MedChemExpress (Shanghai, China).

Ubiquitylation assay

CRC cells were treated with the chemotherapeutic agent DOX and then exposed to 4Gy irradiation for 24h later, then cells were washed with PBS and lysed with protein lysate. Immunoprecipitation was performed by adding 1-1.5μg anti-FOXP4 antibody to the protein lysate, and then western blotting was

performed using anti-Ub to detect FOXP4 ubiquitination. Relevant antibodies were detailed in Supplementary Table S9.

Statistical analysis

Correlations between proteins were illustrated by Pearson correlation analysis, data was detailed in Supplementary Table S10. Relative expression levels of target proteins in IHC, IF and WB experiments were analyzed by Image J software (NIH, USA). Statistical analyses and plots were drawn using Graphpad prism software (version 10.4.1; GraphPad, San Diego, CA, USA). Each set of experiments was repeated three times and two-tailed Student's t-test was used to compare the significance of differences. Statistical significance was defined as $P < 0.05$.
