# Supplementary material for: Patients‐Derived Organoids Sequencing‐based FOXP4 Facilitates Radioresistance by Transcriptionally Modifying GPX4 to Regulate ferroptosis in Colorectal Cancer
Source: Adv Sci (Weinh). 2025 Aug 11;12(37):e07080. doi: 10.1002/advs.202507080 (PMC12499432; doi:10.1002/advs.202507080)

ADVANCED SCIENCE
Open Access

Supporting Information

for *Adv. Sci.*, DOI 10.1002/advs.202507080

Patients-Derived Organoids Sequencing-based FOXP4 Facilitates Radioresistance by Transcriptionally Modifying GPX4 to Regulate ferroptosis in Colorectal Cancer

Qianping Chen, Yuzhao Jin, Luyu Liao, Rong Shen, Xingnan Ge, Qingyu Jiang, Ying Huang, Quanquan Sun, Dong Liu, Luying Liu, Tongxin Liu, Qinghui Dai, Xiyuan Tang, Zhe Han, Xin Gao, Xinhua Lin*, Wei Mao* and Ji Zhu**

Supplemental Figures and Figures Legends

Figure S1. Bioinformatics analysis of colorectal cancer PDOs and cell lines with different radioresistance.

A. Heatmap of Copy number variation (CNV) in rectal cancer driver genes (P, patient tumors; O, organoids). Gene copy numbers were transformed as log2 ratios per gene (blue, gains; red, losses).

B. CCK-8 assay measured cell viability of HCT116, HCT116R, HCT15 and HCT15R cells at 48h after 4Gy IR or non-IR. $^{**}P < 0.01$ and $^{***}P < 0.001$.

C. Volcano plot of DEGs between PDO-R and PDO-S groups, with a fold change ≥ 1.5 or ≤ 0.67 and *P*-value < 0.01.

D. Volcano plot of DEGs between HCT116R and HCT116 cells, with a fold change ≥ 1.5 or ≤ 0.67 and *P*-value < 0.01.

E. Volcano plot of DEGs between HCT15R and HCT15 cells, with a fold change ≥ 1.5 or ≤ 0.67 and *P*-value < 0.01.

F. Volcano plot of DEGs between HCT15 and HCT116 cells, with a fold change ≥ 1.5 or ≤ 0.67 and *P*-value < 0.01.

G. Volcano plot of DEGs between colorectal cancer tissues and adjacent normal tissues from the GEPIA database, with a fold change ≥ 2.0 or ≤ 0.50 and *P*-value < 0.01.

H. Box scatter diagrams of the relative expression level of FOXP4 in tumor and adjacent normal tissues according to GEPIA dataset.

I and J. Kaplan-Meier curves of CRC survivals based on the expression status of FOXP4 gene according to GEPIA dataset (I) and KM plotter (J).

Figure S2. GO enrichment analysis of DEGs across radioresistant and radiosensitive colorectal cancer models.

GO analysis of DEGs from PDO-R vs PDO-S, HCT116R vs HCT116, HCT15R vs HCT15, and HCT15 vs HCT116. Fold change ⩾ 1.5 or ⩽ 0.67, *p*-value < 0.01.

Figure S3. The effect of ferroptosis on radioresistance in colorectal cancer *in vivo*.

A. Relative expression level of GPX4 and 4HNE protein in HCT116, HCT116R, HCT15 and HCT15R cells (see Fig. 3E).

B. CCK-8 assay measured cell viability of HCT116 and HCT15 cells at 48h after 4Gy IR or Erastin treatment.

C. Cell-titer assay measured cell viability of PDOs at 5d after 8Gy irradiation or Erastin treatment (see Fig. 3G).

D. Representative IHC images (×20) and quantitative results of FOXP4 and 4HNE protein expression in PDOX xenograft tumors. Scale bar, 50μm.

E. Representative IHC images (×20) and quantitative results of FOXP4 and 4HNE protein expression in nude mice xenograft tumors. Scale bar, 50μm.

F. Pattern plots of nude mice treated with Erastin that were irradiated at 8Gy*3d and then executed at the appropriate time.

G. Tumor volume of Erastin, IR and Erastin+IR groups was examined every 3 days until 9 days after IR.

H. General view of tumor mass of each indicated group at 9 days after IR.

*$P < 0.05$, **$P < 0.01$, ***$P < 0.001$ and ****$P < 0.0001$.

Figure S4. FOXP4 modulated ferroptosis in CRC cells.

A. Relative expression level of FOXP4, GPX4 and 4HNE protein in HCT116 and HCT15 cells (see Fig. 4B).

B. Quantification of the relative fluorescence intensity of ROS in HCT116 and HCT15 cells transfected with shNC or shFOXP4 at 4h after 4 Gy IR.

C. Quantification of the relative fluorescence intensity of liperfluo in HCT116 and HCT15 cells transfected with shNC or shFOXP4 at 4h after 4 Gy IR.

D. Quantification of the relative fluorescence intensity of MDA in HCT116 and HCT15 cells transfected with shNC or shFOXP4 at 4h after 4 Gy IR.

E. Quantitative results of FOXP4, 4HNE and GPX4 protein expression (see Fig. 4E) of nude mice xenograft tumors.

F and G. Quantification of the relative fluorescence intensity of MDA in HCT116 and HCT15 cells transfected with shNC or shFOXP4 and treated with Fer-1 simultaneously at 4h after 4Gy IR.*$P < 0.05$, **$P < 0.01$, ***$P < 0.001$ and ****$P < 0.0001$.

Figure S5. Time-course analysis of FOXP4 and GPX4 expression and promoter activity in CRC cells following ionizing radiation.

A. Representative images and quantitative results of FOXP4, GPX4 and ACTIN protein expression in HCT116 and HCT15 cells.

B. Dual luciferase reporter analysis showed the transcription of GPX4 in HCT116 and

HCT15 cells after 4 Gy IR.

C. qPCR assay showed the mRNA level of GPX4 at 4,8,12,24 and 48h in HCT116 and HCT15 cells after 4Gy IR.

n = 3, **P* < 0.05, ***P* < 0.01, ****P* < 0.001 and *****P* < 0.0001.

Figure S6. DOX affected the radiotherapy efficacy in CRC *in vivo*.

A. Quantification data of ChIP PCR (see Fig. 6F) in HCT116 and HCT15 cells with overexpression of FOXP4-WT or FOXP4-FH-Del; n = 3. ***P* < 0.01 and ****P* < 0.001.

B. Cartoon illustration showed the molecular structures of Doxorubicin, Resveratrol, Valproic acid and Afimoxif.

C. Box scatter diagrams showed the IC50 of DOX distribution in different tissue types.

D. Pattern plots of nude mice treated with DOX that were irradiated at 8Gy*3d and then executed at the appropriate time.

E. Tumor volume of DOX, IR and DOX+IR groups was examined every 3 days until 9 days after IR (8Gy*3d). ****P* < 0.001.

F. General view of tumor mass of each indicated group at 9 days after IR (8Gy*3d).

G. qPCR assay showed the mRNA level of FOXP4 in HCT15 cells after DOX treatment.

H. Quantification of western blot assay of FOXP4 proteins in HCT15 cells at 4h after 4Gy IR. MG-132 (10μM) or CQ (50μM) was added before IR (see Fig.7J). ****P* < 0.001.

I. Quantification of western blot assay of Ub in HCT15 cells at 4h after 4Gy IR (see Fig. 7K). ****P* < 0.001.

Supplementary Figure S1

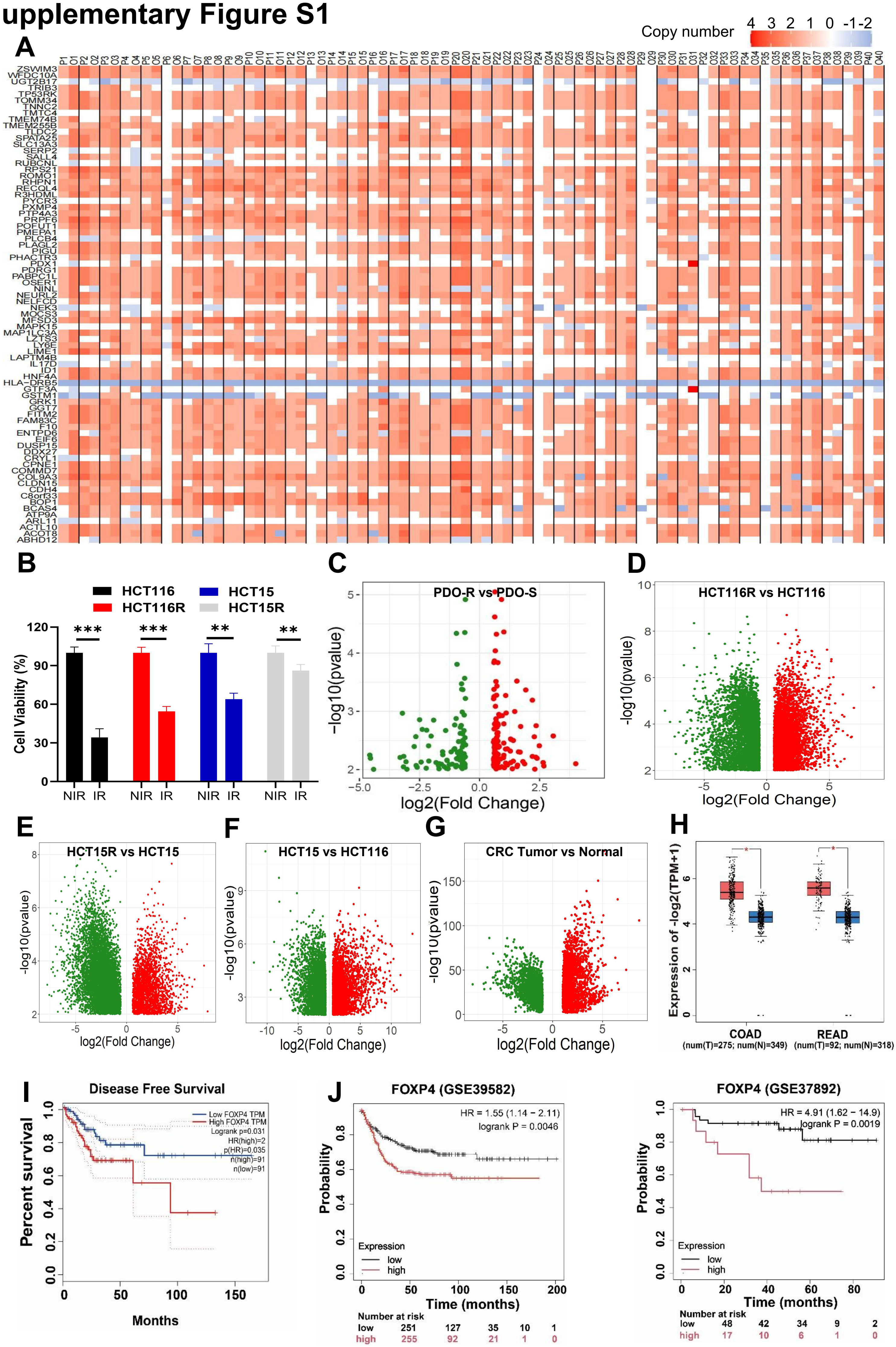

Supplementary Figure S2

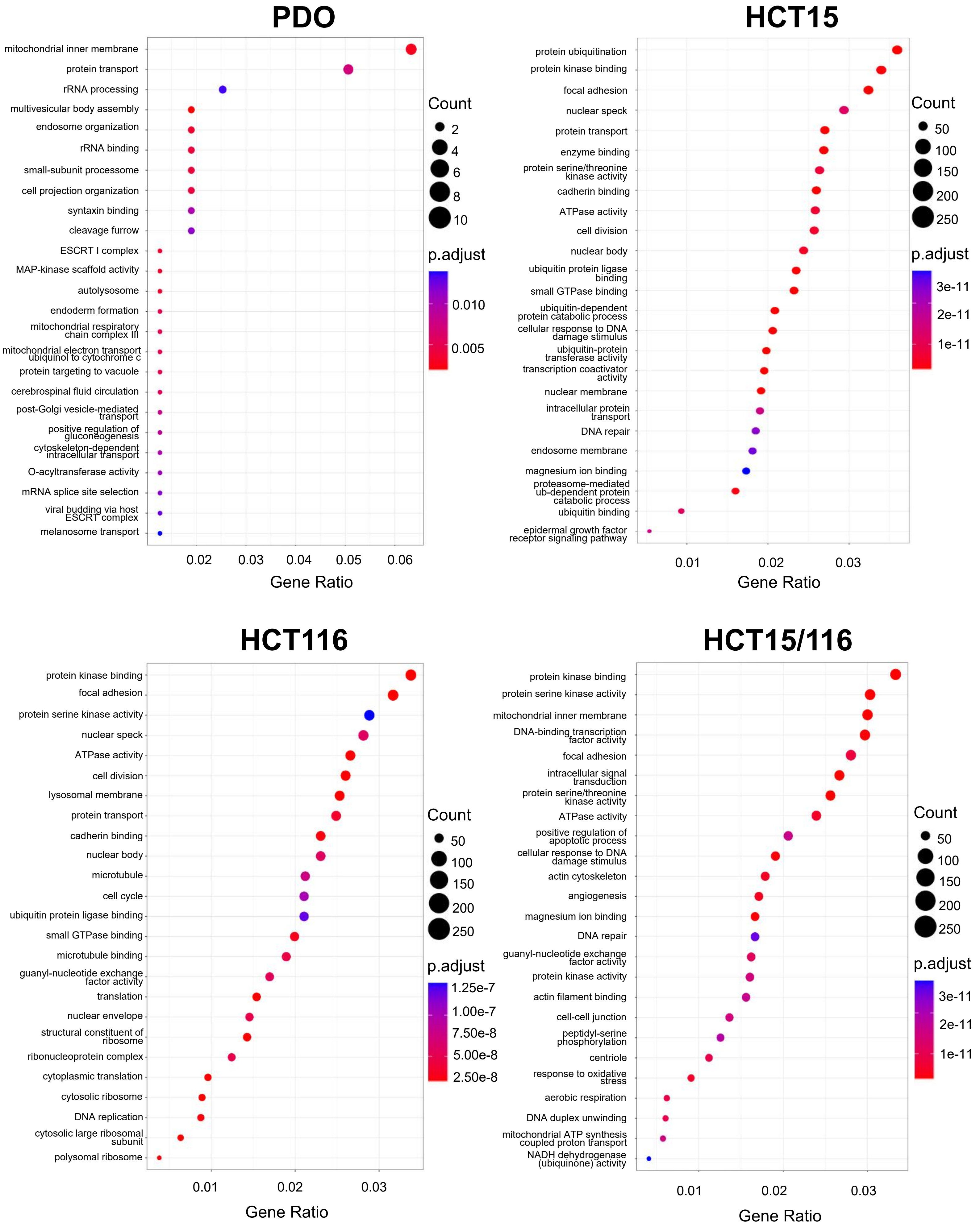

Supplementary Figure S3

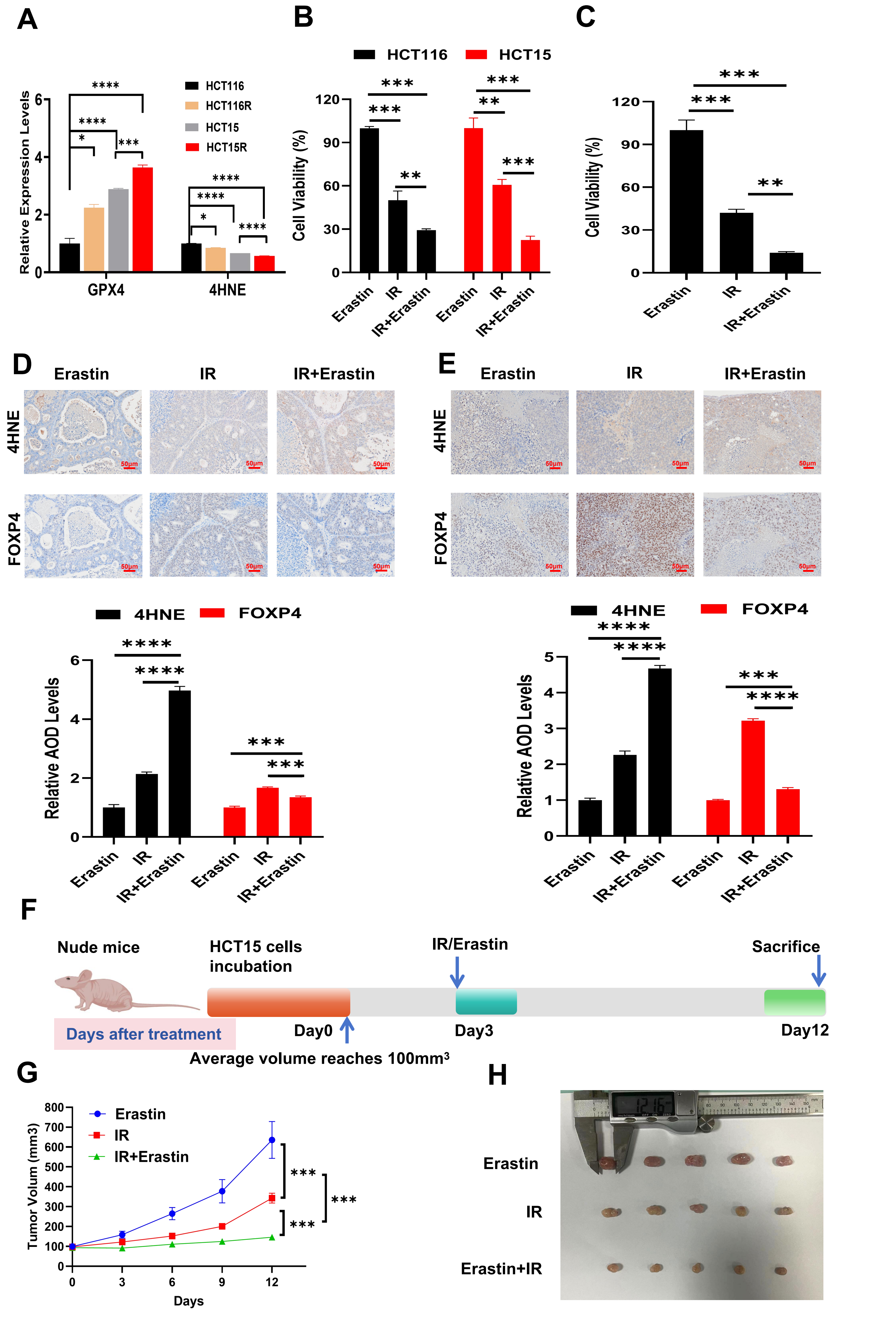

Supplementary Figure S4

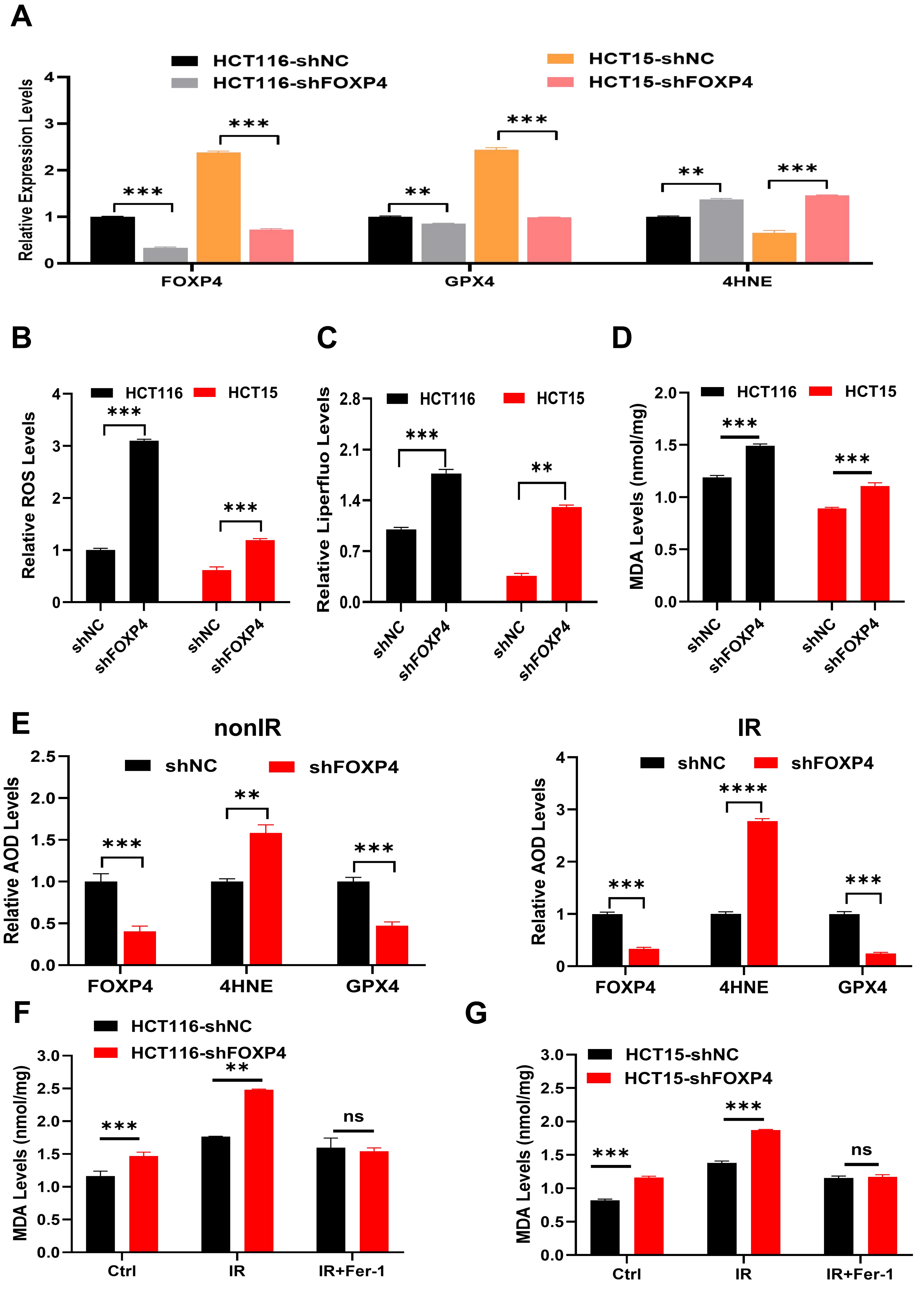

Supplementary Figure S5

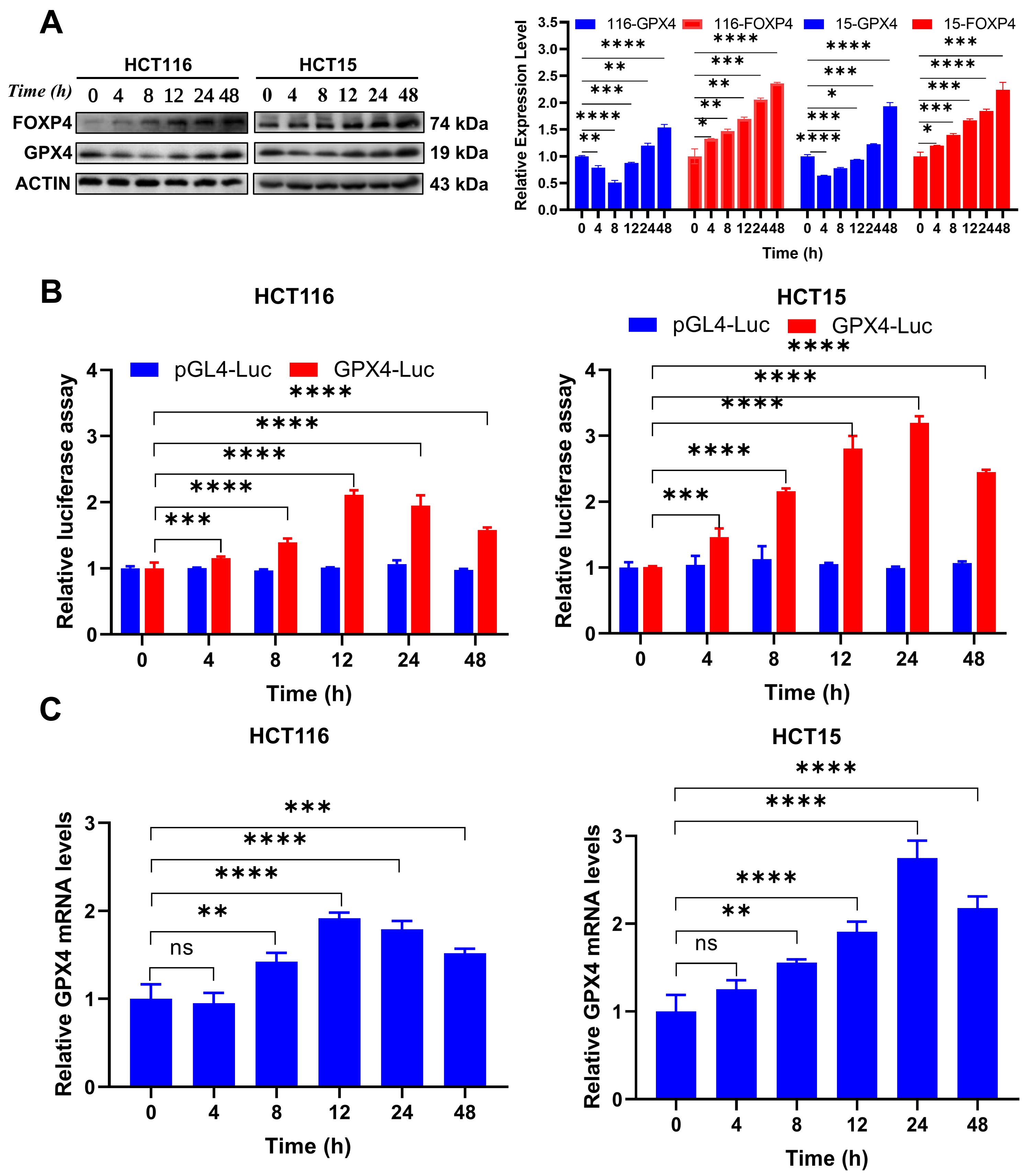

Supplementary Figure S6

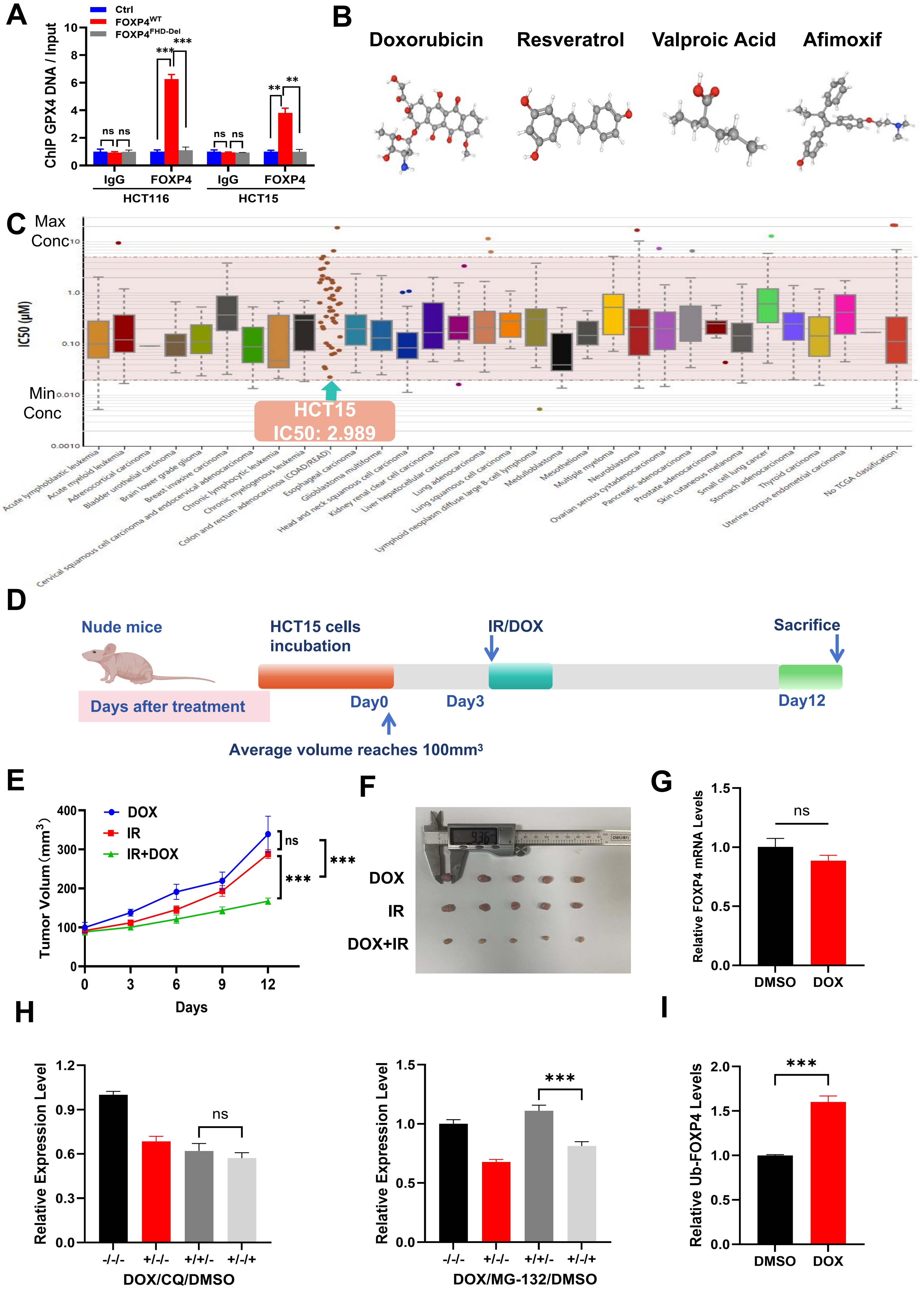

Supplement: Supplementary file 2 — Supporting Information [file ADVS-12-e07080-s002.pdf]
