# Supplementary material for: Patients‐Derived Organoids Sequencing‐based FOXP4 Facilitates Radioresistance by Transcriptionally Modifying GPX4 to Regulate ferroptosis in Colorectal Cancer
Source: Adv Sci (Weinh). 2025 Aug 11;12(37):e07080. doi: 10.1002/advs.202507080 (PMC12499432; doi:10.1002/advs.202507080)
Supplement: Supplementary file 11 — Supporting Information [file ADVS-12-e07080-s003.docx]

**Table S9. List of antibodies**

| **Antibody** | **Manufacturer** | **Catalog** | **Dilution** | **Assay** |
| --- | --- | --- | --- | --- |
| anti-rabbit FOXP4 | Proteintech Group  (Shanghai, China) | 16772-1-AP | 1:1000  1:100  1:400 | WB  CUT&TAG  IHC |
| anti-rabbit GPX4 | Abcam Technology  (MA, USA) | ab125066 | 1:1000  1:1500 | WB  IHC |
| anti-rabbit SLC7A11 | Cell signaling Technology  (TX, USA) | 12691S | 1:1000 | WB |
| anti-rabbit 4-HNE | Abcam Technology  (MA, USA) | ab46545 | 1:1000  1:400 | WB  IHC |
| anti-rabbit β-actin | HuaBio, Inc.  (Hangzhou, China) | EM21002 | 1:2000 | WB |
| anti-mouse α-tubulin | Sigma-Aldrich  (MO, USA) | T9026 | 1；2000 | WB |
| anti‐rabbit IgG | Cell signaling Technology  (TX, USA) | 3423S | 1:100 | Co-IP |
| anti-rabbit Ub | Proteintech Group  (Shanghai, China) | 10201-2-AP | 1:1000 | WB |
| anti-rabbit Ki67 | BD Biosciences  (CA, USA) | 550609 | 1:500 | IHC |
| anti-rabbit CDX2 | Abcam Technology  (MA, USA) | Ab7753 | 1:500 | IHC |
| anti-rabbit β-catenin | BD Biosciences  (CA, USA) | 610153 | 1:100 | IHC |
| anti-rabbit CK20 | Abcam Technology  (MA, USA) | Ab76126 | 1:250 | IHC |
| anti-rabbit CK-pan | Abcam Technology  (MA, USA) | Ab76541 | 1:250 | IHC |
| anti-rabbit FOXP1 | Cell signaling Technology  (TX, USA) | 4402S | 1:1000 | WB |
| anti-rabbit FOXP2 | Abcam Technology  (MA, USA) | Ab16046 | 1:1000 | WB |
