# Supplementary material for: Patients‐Derived Organoids Sequencing‐based FOXP4 Facilitates Radioresistance by Transcriptionally Modifying GPX4 to Regulate ferroptosis in Colorectal Cancer
Source: Adv Sci (Weinh). 2025 Aug 11;12(37):e07080. doi: 10.1002/advs.202507080 (PMC12499432; doi:10.1002/advs.202507080)
Supplement: Supplementary file 15 — Supporting Information [file ADVS-12-e07080-s011.pptx]

## Slide 1
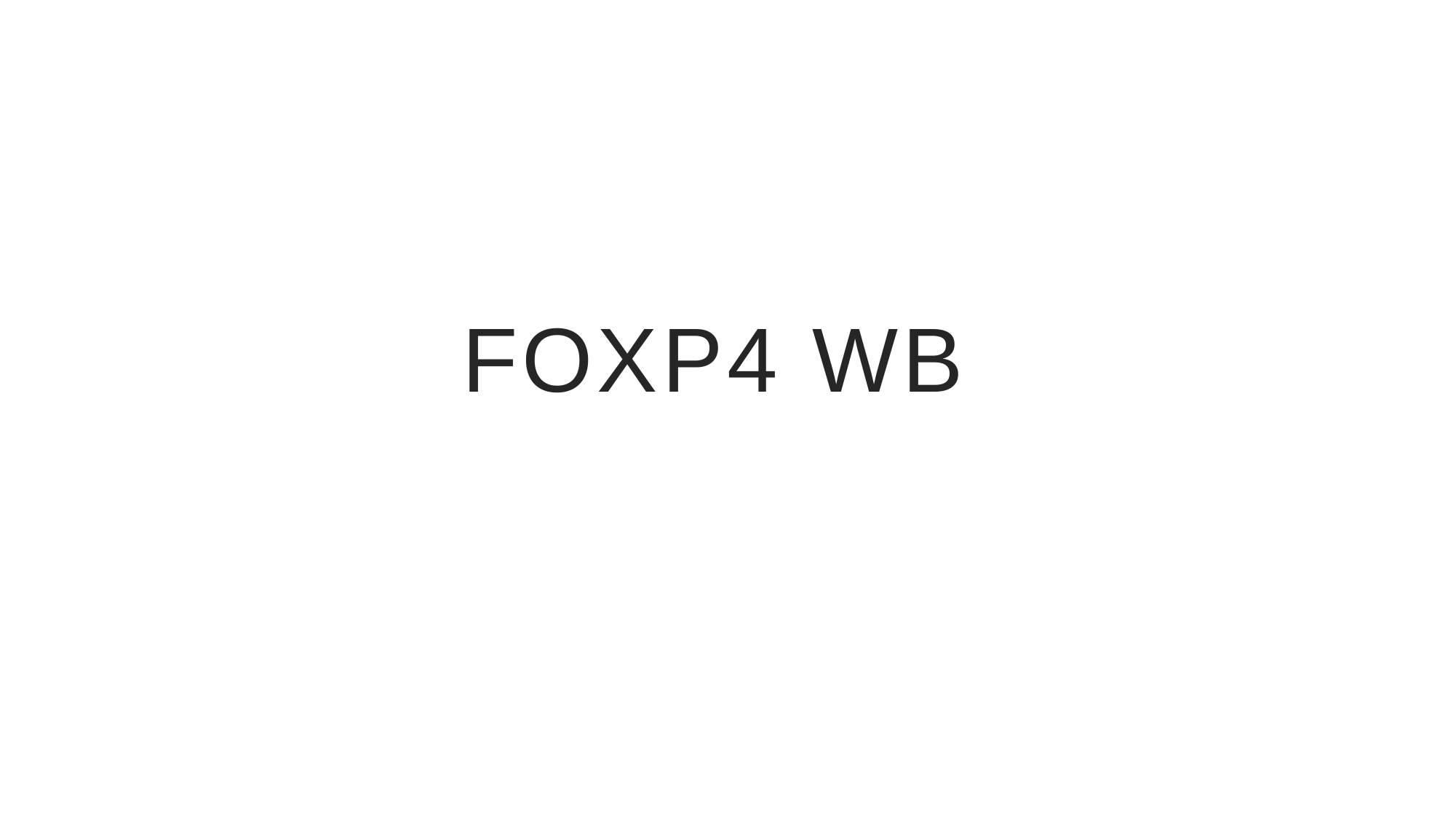

# FOXP4 WB

## Slide 2
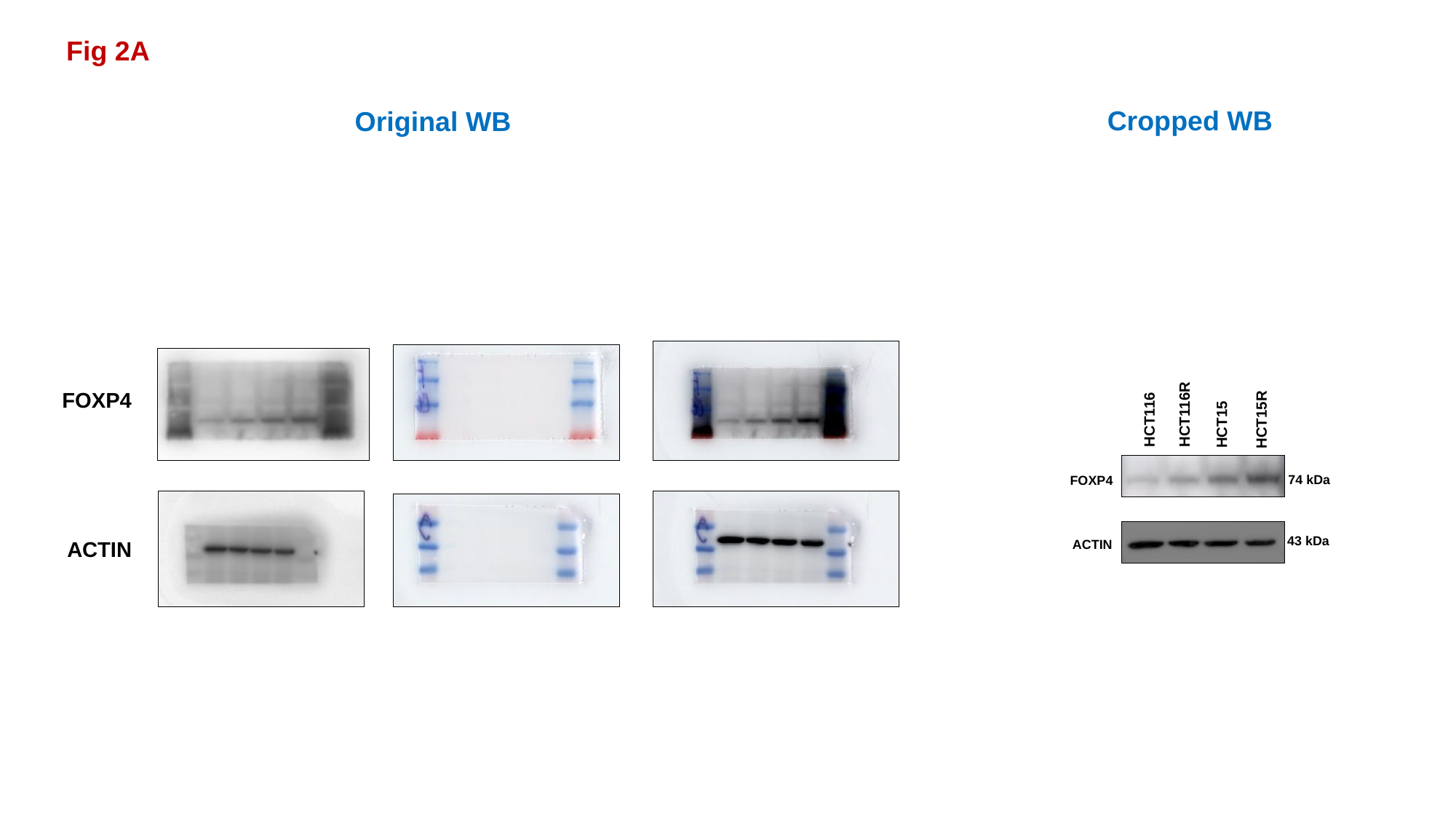

Fig 2A
Cropped WB
Original WB
HCT116R
HCT116
HCT15R
HCT15
74 kDa
FOXP4
43 kDa
ACTIN
FOXP4
ACTIN

## Slide 3
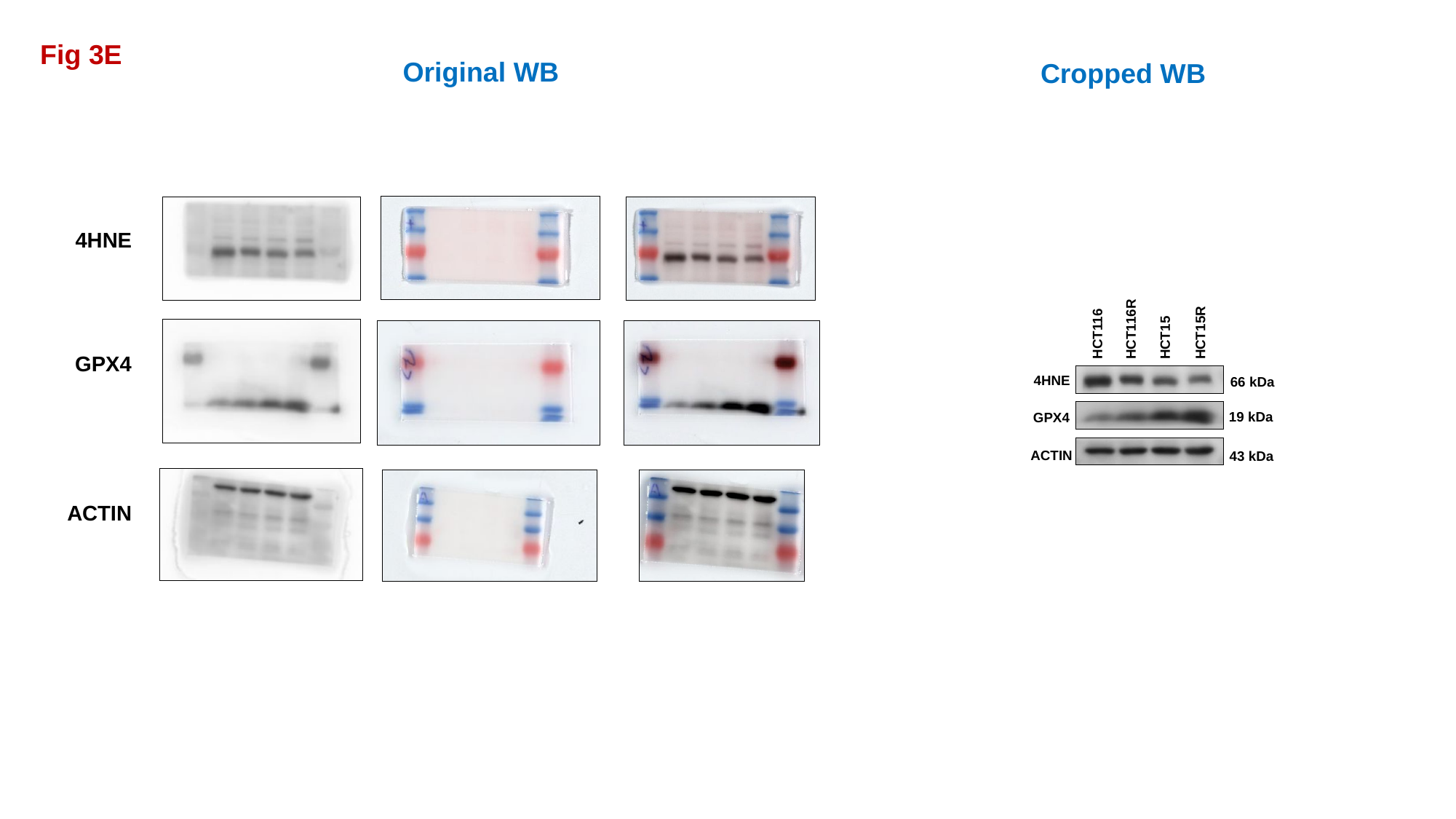

Fig 3E
Original WB
Cropped WB
4HNE
HCT15R
HCT116
HCT15
66 kDa
19 kDa
ACTIN
43 kDa
4HNE
GPX4
HCT116R
GPX4
ACTIN

## Slide 4
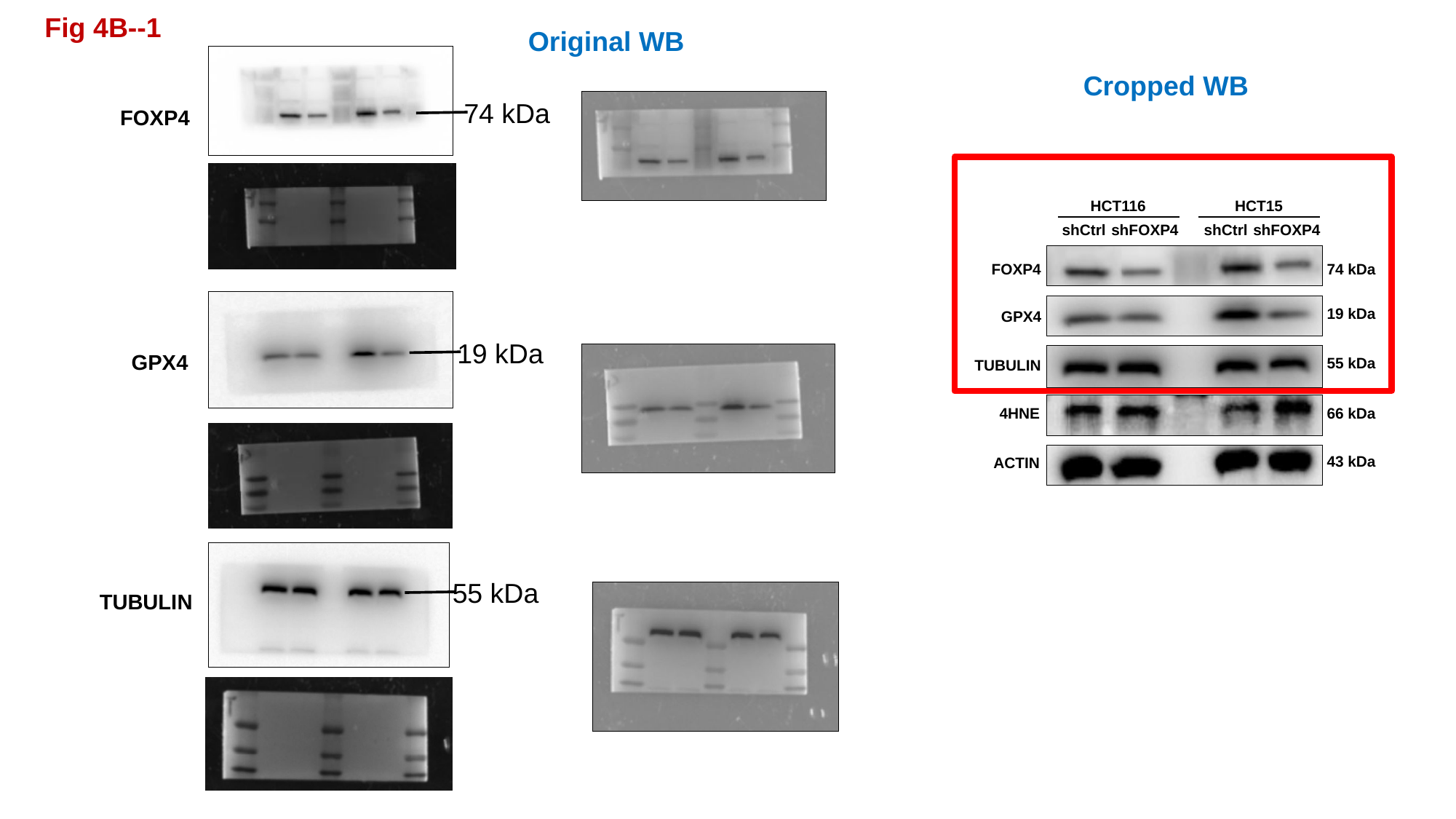

Original WB
Fig 4B--1
Original WB
Cropped WB
74 kDa
FOXP4
HCT116
HCT15
shCtrl
shFOXP4
shCtrl
shFOXP4
FOXP4
74 kDa
19 kDa
GPX4
19 kDa
GPX4
55 kDa
TUBULIN
4HNE
66 kDa
43 kDa
ACTIN
55 kDa
TUBULIN

## Slide 5
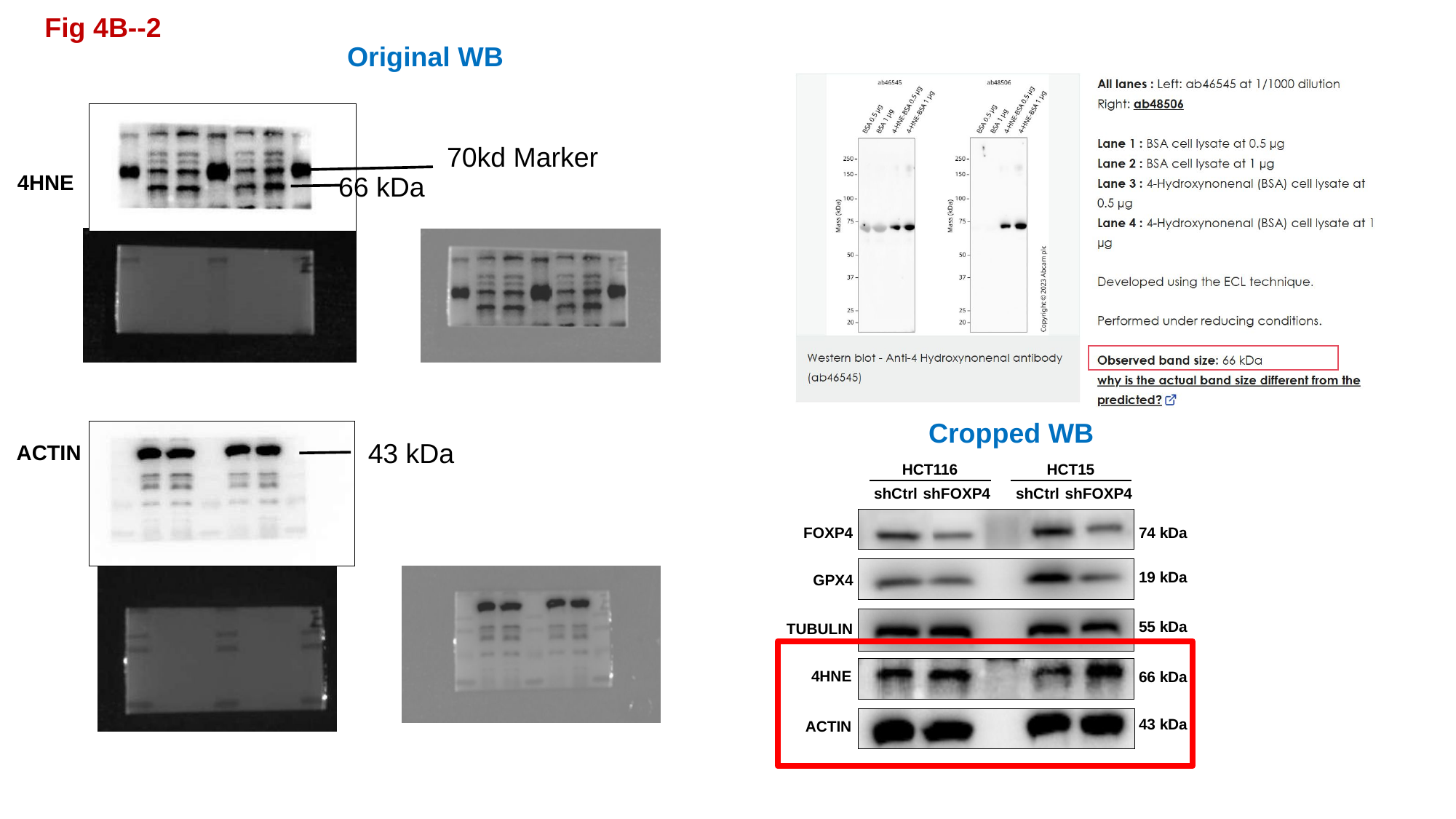

Fig 4B--2
Original WB
70kd Marker
4HNE
66 kDa
Cropped WB
43 kDa
ACTIN
HCT116
HCT15
shCtrl
shFOXP4
shCtrl
shFOXP4
FOXP4
74 kDa
19 kDa
GPX4
55 kDa
TUBULIN
4HNE
66 kDa
43 kDa
ACTIN

## Slide 6
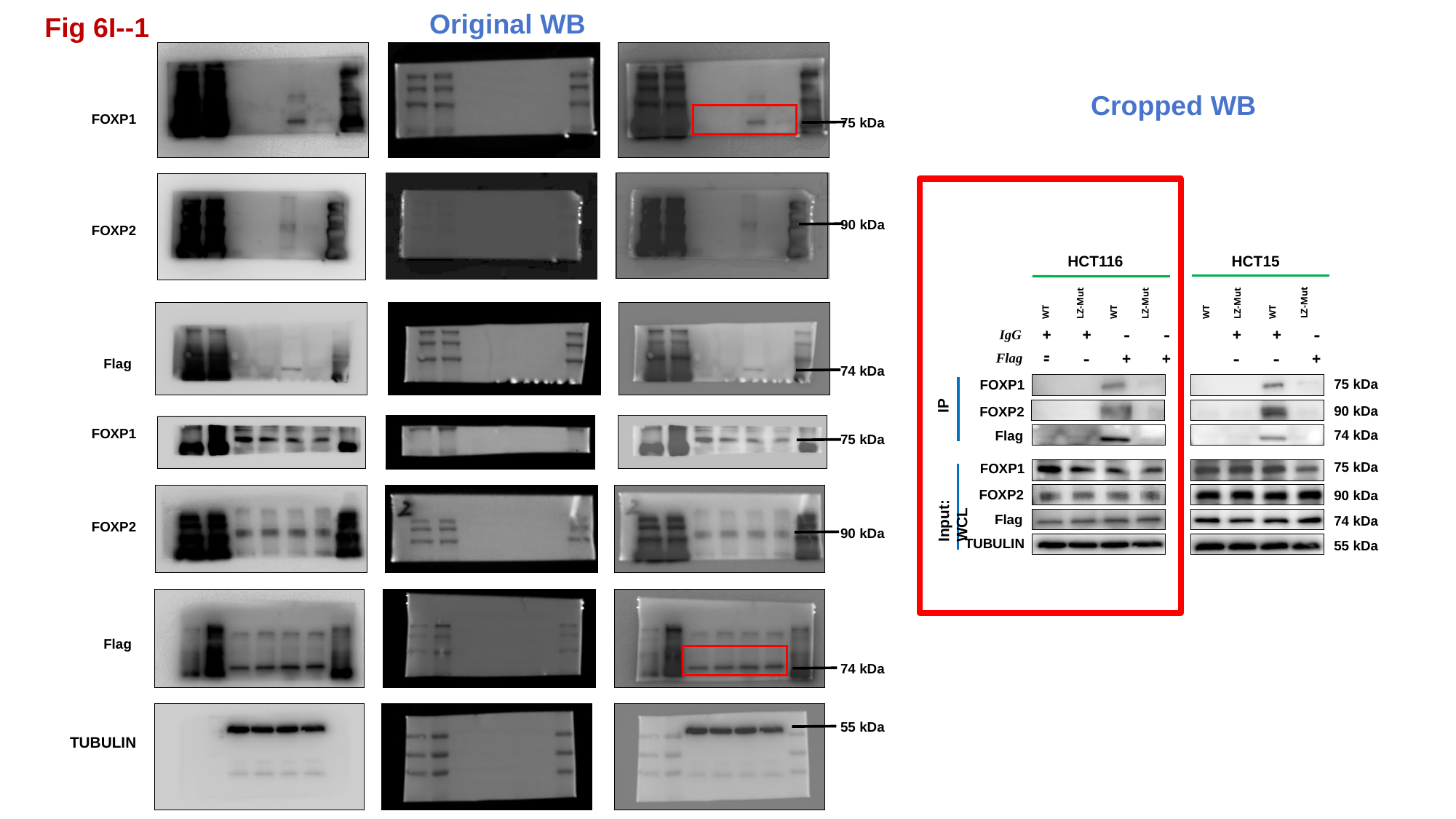

Original WB
Cropped WB
FOXP1
75 kDa
90 kDa
FOXP2
HCT116 HCT15
LZ-Mut
LZ-Mut
LZ-Mut
LZ-Mut
WT
WT
WT
WT
+ + - - + + - -
IgG
- - + + - - + +
Flag
Flag
74 kDa
75 kDa
FOXP1
IP
90 kDa
FOXP2
FOXP1
74 kDa
Flag
75 kDa
75 kDa
FOXP1
Input: WCL
FOXP2
90 kDa
Flag
74 kDa
FOXP2
90 kDa
TUBULIN
55 kDa
Flag
74 kDa
55 kDa
TUBULIN
Fig 6I--1

## Slide 7
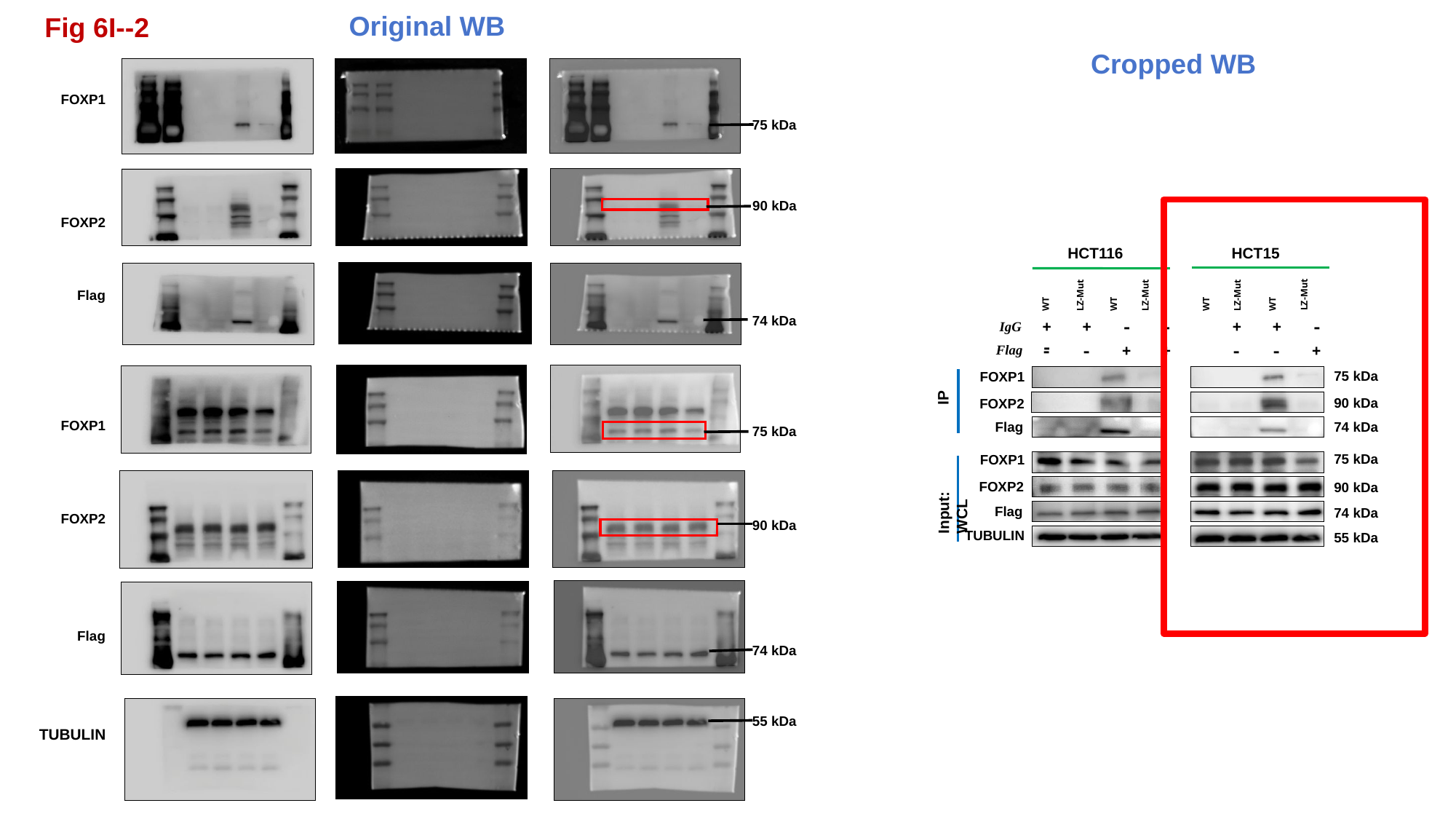

Original WB
Cropped WB
FOXP1
FOXP2
Flag
FOXP1
FOXP2
Flag
TUBULIN
75 kDa
90 kDa
HCT116 HCT15
LZ-Mut
LZ-Mut
LZ-Mut
LZ-Mut
WT
WT
WT
WT
+ + - - + + - -
75 kDa
90 kDa
74 kDa
75 kDa
90 kDa
74 kDa
55 kDa
FOXP1
IP
FOXP2
Flag
FOXP1
Input: WCL
FOXP2
Flag
TUBULIN
IgG
Flag
- - + + - - + +
74 kDa
75 kDa
90 kDa
74 kDa
55 kDa
Fig 6I--2

## Slide 8
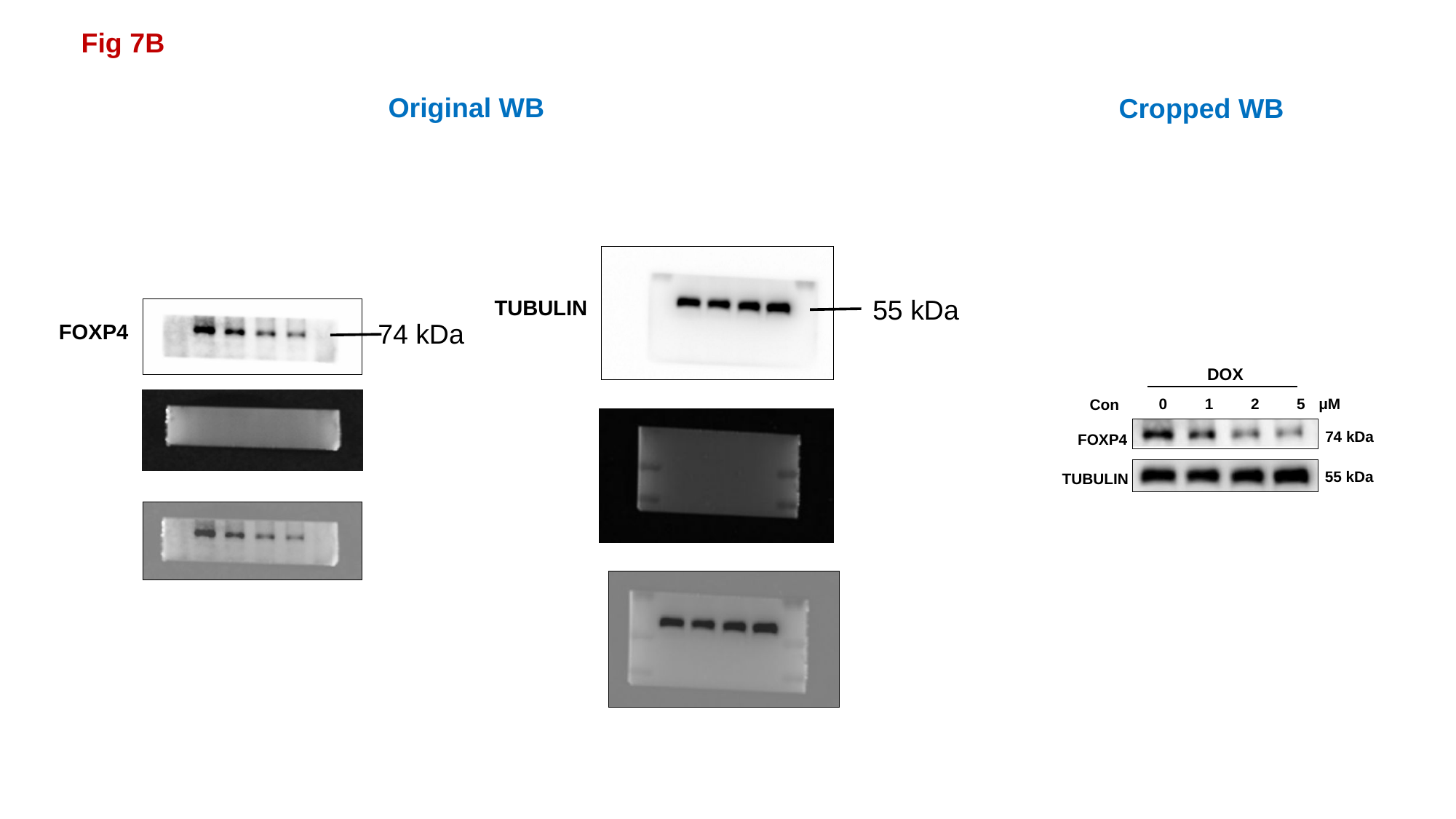

Fig 7B
Original WB
Cropped WB
55 kDa
TUBULIN
74 kDa
FOXP4
DOX
 0 1 2 5
μM
74 kDa
FOXP4
55 kDa
TUBULIN
Con

## Slide 9
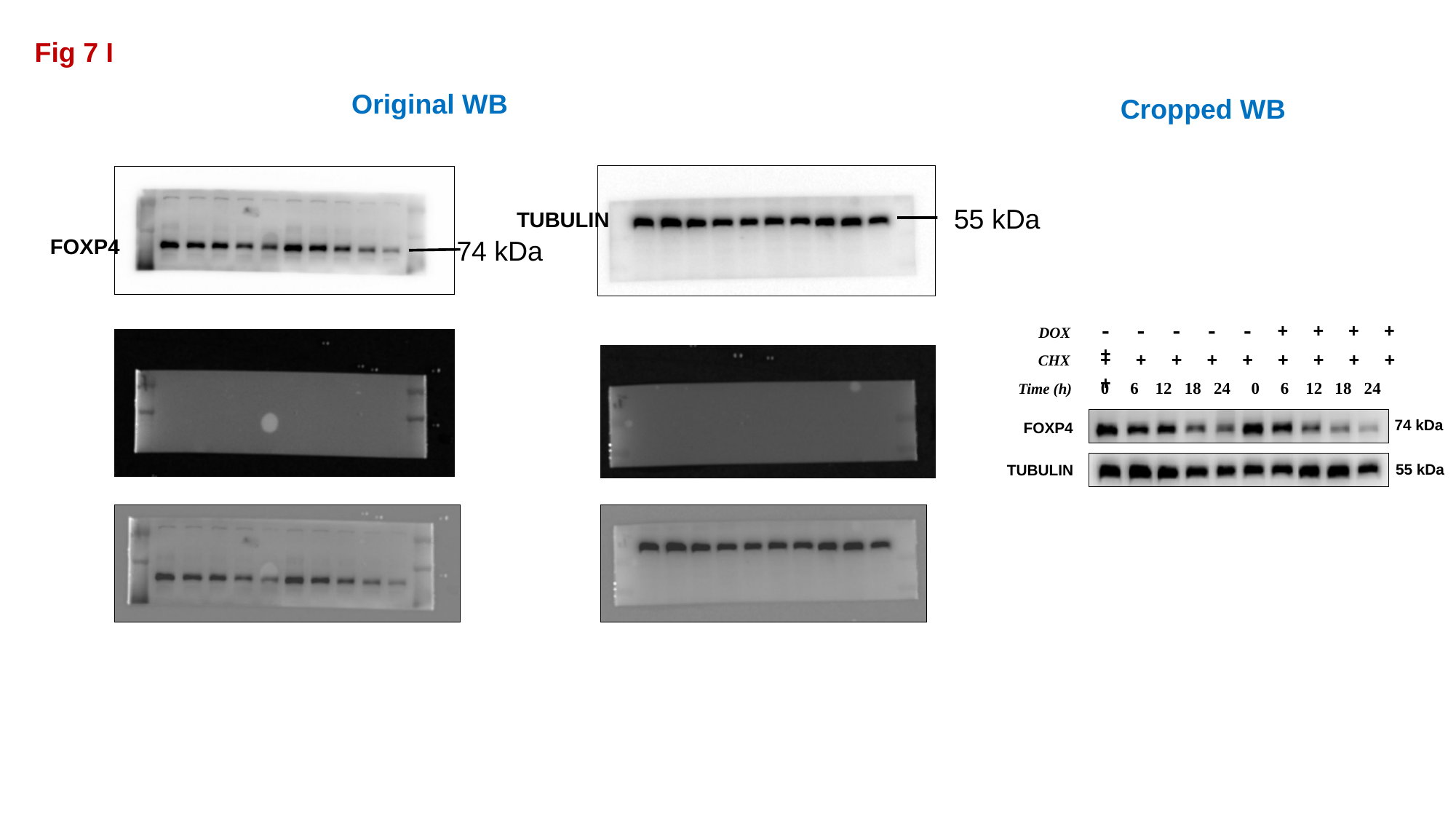

Fig 7 I
Original WB
Cropped WB
55 kDa
TUBULIN
FOXP4
74 kDa
- - - - - + + + + +
DOX
+ + + + + + + + + +
CHX
0 6 12 18 24 0 6 12 18 24
Time (h)
74 kDa
FOXP4
55 kDa
TUBULIN

## Slide 10
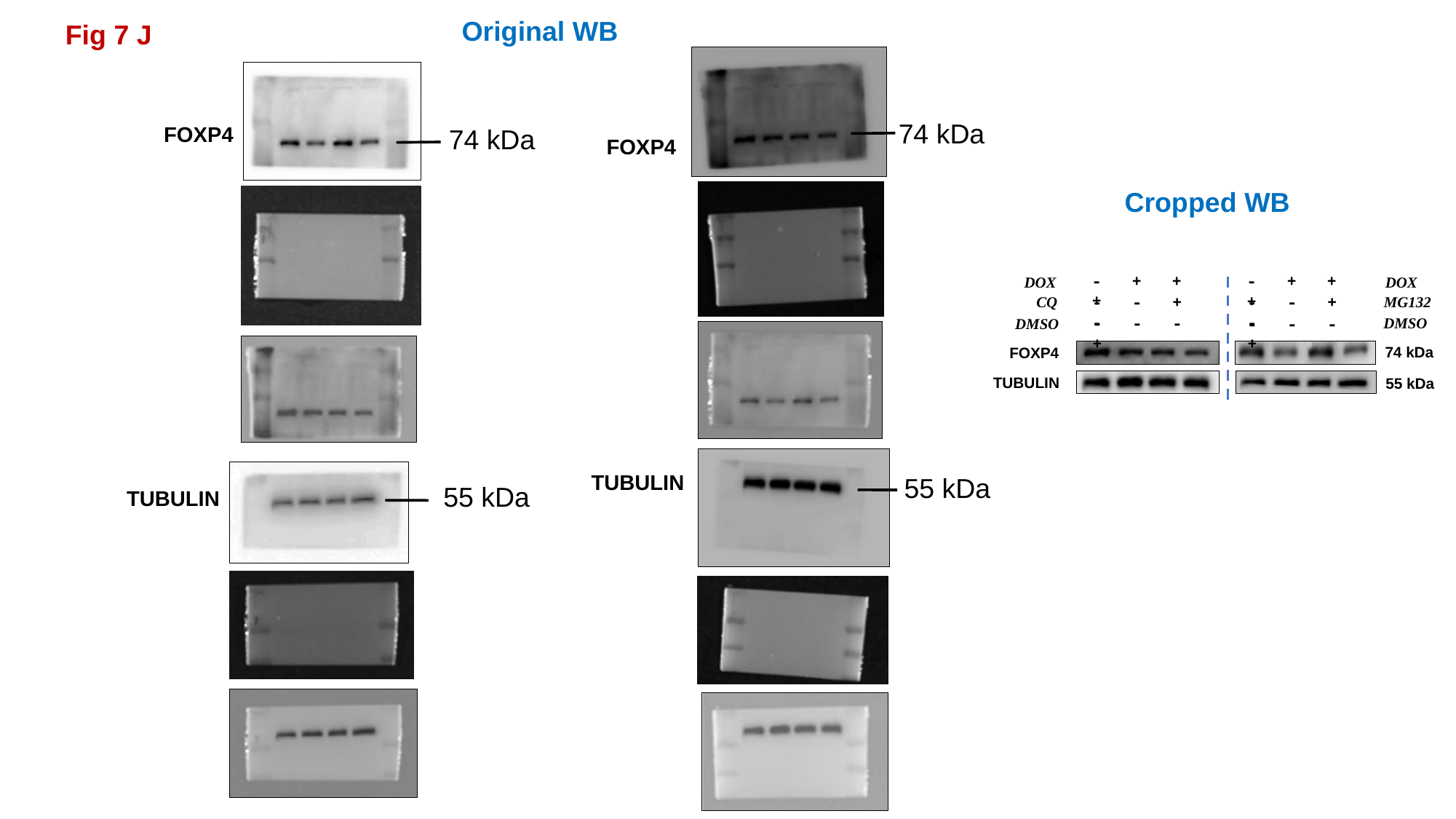

Original WB
Fig 7 J
74 kDa
FOXP4
74 kDa
FOXP4
Cropped WB
- + + +
- + + +
DOX
DOX
- - + -
- - + -
CQ
MG132
- - - +
- - - +
DMSO
DMSO
74 kDa
FOXP4
TUBULIN
55 kDa
TUBULIN
55 kDa
55 kDa
TUBULIN

## Slide 11
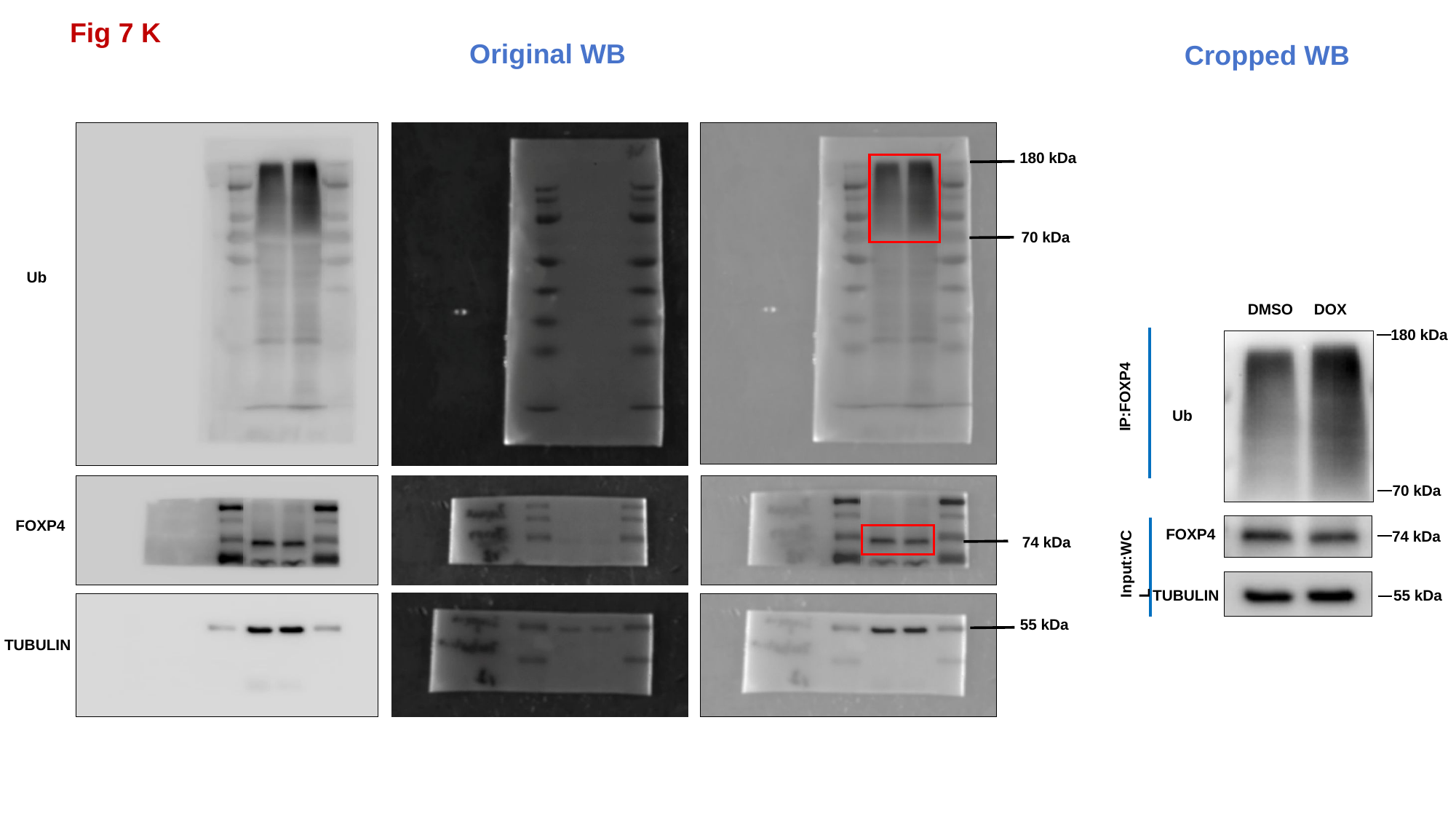

Fig 7 K
Original WB
Cropped WB
180 kDa
70 kDa
Ub
DMSO DOX
180 kDa
IP:FOXP4
Ub
70 kDa
Input:WCL
FOXP4
74 kDa
TUBULIN
55 kDa
FOXP4
74 kDa
55 kDa
TUBULIN

## Slide 12
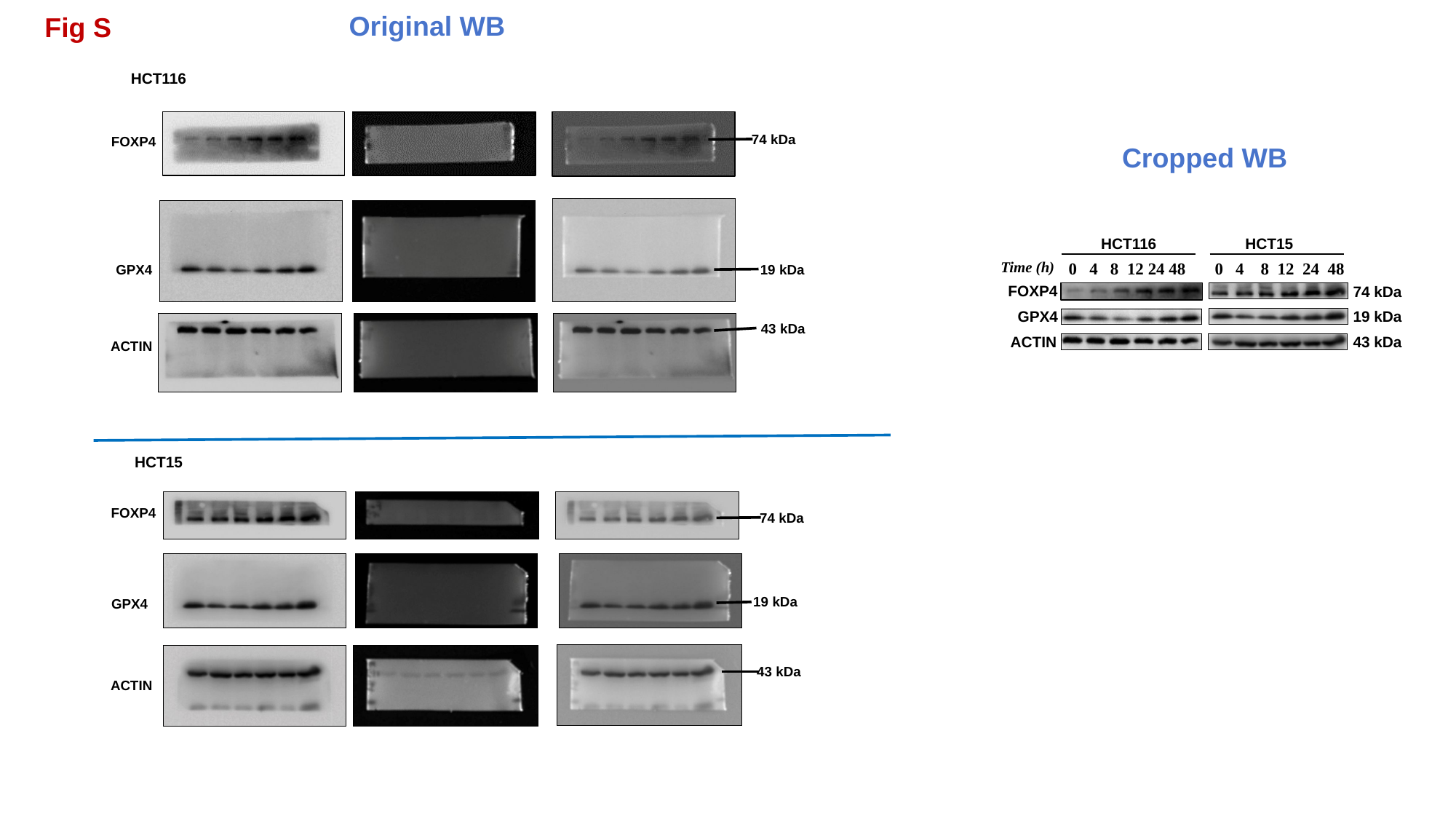

Original WB
Fig S
HCT116
74 kDa
FOXP4
Cropped WB
HCT116
HCT15
Time (h)
0 4 8 12 24 48 0 4 8 12 24 48
74 kDa
19 kDa
43 kDa
FOXP4
GPX4
ACTIN
GPX4
19 kDa
43 kDa
ACTIN
HCT15
FOXP4
74 kDa
19 kDa
GPX4
43 kDa
ACTIN
